# Probing the role of the residues in the active site of the transaminase from *Thermobaculum terrenum*

**DOI:** 10.1371/journal.pone.0255098

**Published:** 2021-07-29

**Authors:** Ekaterina Yu. Bezsudnova, Alena Yu. Nikolaeva, Alina K. Bakunova, Tatiana V. Rakitina, Dmitry A. Suplatov, Vladimir O. Popov, Konstantin M. Boyko

**Affiliations:** 1 Bach Institute of Biochemistry, Research Center of Biotechnology of the Russian Academy of Sciences, Moscow, Russian Federation; 2 Shemyakin & Ovchinnikov Institute of Bioorganic Chemistry of the Russian Academy of Sciences, Moscow, Russian Federation; 3 Lomonosov Moscow State University, Belozersky Institute of Physicochemical Biology, Moscow, Russian Federation; Weizmann Institute of Science, ISRAEL

## Abstract

Creating biocatalysts for (*R*)-selective amination effectively is highly desirable in organic synthesis. Despite noticeable progress in the engineering of (*R*)-amine activity in pyridoxal-5’-phosphate-dependent transaminases of fold type IV, the specialization of the activity is still an intuitive task, as there is poor understanding of sequence-structure-function relationships. In this study, we analyzed this relationship in transaminase from *Thermobaculum terrenum*, distinguished by expanded substrate specificity and activity in reactions with L-amino acids and (*R*)-(+)-1-phenylethylamine using α-ketoglutarate and pyruvate as amino acceptors. We performed site-directed mutagenesis to create a panel of the enzyme variants, which differ in the active site residues from the parent enzyme to a putative transaminase specific to (*R*)-primary amines. The variants were examined in the overall transamination reactions and half-reaction with (*R*)-(+)-1-phenylethylamine. A structural analysis of the most prominent variants revealed a spatial reorganization in the active sites, which caused changes in activity. Although the specialization to (*R*)-amine transaminase was not implemented, we succeeded in understanding the role of the particular active site residues in expanding substrate specificity of the enzyme. We showed that the specificity for (*R*)-(+)-1-phenylethylamine in transaminase from *T*. *terrenum* arises without sacrificing the specificity for L-amino acids and α-ketoglutarate and in consensus with it.

## Introduction

Transaminases (TAs; aminotransferases; EC 2.6.1.) are pyridoxal-5’-phosphate (PLP)-dependent enzymes that catalyze the reversible stereoselective transfer of an amino group from an amino substrate to a keto acid or ketone, thereby producing a chiral amine or amino acid and a new keto compound [1–3]. Transamination is a double displacement process comprising two half-reactions: (i) the transfer of an amino group from amine or amino acid to the PLP cofactor producing pyridoxamine 5′-phosphate (PMP) and ketone or keto acid and (ii) the stereoselective transfer of an amino group from PMP to another ketone or keto acid restoring the PLP form of the cofactor and producing a new amine or amino acid [1, 4]. Since 2000, TAs have been at the verge of breakthrough biotechnological research encompassing the synthesis of optically pure amines from keto precursors, cascade processes, and whole-cell catalysis [5, 6]. The industrial synthesis of the anti-diabetic drug Sitagliptin and a component of cardiovascular treatment Sacubitril is among the successful application of the TAs [7, 8].

Three families represent PLP-dependent TAs of fold type IV: D-amino acid aminotransferases (DAATs), (*R*)-selective amine transaminases (R-TAs), and branched-chain L-amino acid transaminases (BCATs) (S1 Fig) [9–12]. TAs exhibit high structural similarity to each other; the functional unit of all the investigated TAs is a dimer that contains two active sites that are symmetrically formed by the residues of both subunits [12, 13]. Substrate specificity and strict (*R*)-selectivity or (*S*)-selectivity of the PLP-dependent TAs of fold type IV are the result of different amino acid compositions within similarly organized active sites [12, 14–16]. Generally, the active site is a combination of two pockets: the O-pocket on the phenolic side of the PLP and the P-pocket on the phosphate group side of the PLP [14, 17–19] (S2 Fig). The covalently bound with the catalytic lysine PLP molecule is positioned at the bottom of the pockets, opposite the active site entrance [13]. The contribution of residues from the same positions to the substrate coordination differs among the families and is a key to understanding the *sequence-structure-function* relationship in TAs [13, 14].

DAATs catalyze transamination between D-amino acids and a-ketoglutarate as the amino acceptor, thereby producing keto acids and D-glutamic acid [20, 21]. In DAATs catalysis, the α-COOH group is an important recognition moiety of the substrate: it is coordinated in the O-pocket by the "carboxylate trap" formed by the residues from βX-strand and the O-pocket loop [13, 22]. The other part of the substrate is accommodated in the P-pocket. R-TAs catalyze the deamination of (*R*)-primary amines (that lack α-COOH group) using pyruvate as an amino acceptor [11, 23]. The α-COOH group of pyruvate binds in the O-pocket via the coordination with the only arginine residue located on the O-pocket loop [18, 24]. The latter in R-TAs is longer than in DAATs and BCATs [18, 24, 25]. The P-pocket in R-TAs is small and hydrophobic and limits the substrate spectrum of natural R-TAs to the amines/ketones containing the methyl group.

BCATs catalyze transamination between L-branched-chain amino acids (BCAA) and α-ketoglutarate, producing keto acids and L-glutamic acid [9, 26, 27]. The proL-binding of keto acids in BCATs contrasts the proD-binding of keto acids and ketones in DAATs and R-TAs [12, 28]. In BCATs, the α-COOH group of substrates binds in the P-pocket, which is strictly designed to accommodate this group [13, 19, 28, 29]. The O-pocket is more extensive, less conserved, and formed by the alternating hydrophobic and hydrophilic residues. Despite the lack of charged patches, the binding of the hydrophobic groups in the P-pocket is unfavorable, and (*R*)-primary amines are poor substrates for BCATs (according to the CIP nomenclature (*R*)-primary amines result from L-amino acids by substituting the α-COOH group with an alkyl group) [26, 27, 30].

Over the last decade, TAs with an expanded substrate specificity have been described [13]. Some of them demonstrate properties of both DAATs and R-TAs and transfer the amino group from D-amino acids or (*R*)-primary amines to keto acids, producing new D-amino acids and ketones [16]. Others exhibit properties of both BCATs and R-TAs and transfer the amino group from L-amino acids and (*R*)-primary amines to keto acids, producing new L-amino acids and ketones [13, 15, 31]. TAs with an expanded substrate specificity differ in the active sites from the canonical DAATs, R-TAs, and BCATs [13, 15, 16, 31]. TA from *Thermobaculum terrenum* (*TaTT)* converts BCAA and L-aromatic amino acids at a high rate [15] and catalyzes the transfer of the amino group from ((*R*)-(+)-1-phenylethylamine (R-PEA) to α-ketoglutarate producing acetophenone and L-glutamic acid at a rate similar to that of canonical R-TAs (S1 Fig) [14, 15, 18, 23]. The functional dimer of *TaTT* is close to the dimers of known BCATs; however, the P-pocket comprises only one site for the α-COOH group binding, and the O-pocket is enriched with hydrophobic residues, which disturb the alternation of hydrophobic and hydrophilic residues typical of BCATs. These changes appear to favor the binding of substrates with the aromatic moiety.

In the current paper, we used a combination of bioinformatics approaches, site-directed mutagenesis, and kinetic and structural analyses to estimate the role of the residues in the *TaTT* active site in achieving its expanded substrate specificity and to improve the *TaTT* specificity toward (*R*)-primary amines. Previously, Voss et al. succeeded in introducing R-TА-like activity into DAAT from *Bacillus subtilis* but failed to generate R-TA-like activity on the scaffold of canonical BCAT from *Escherichia coli* [32]. We suggested that *TaTT* as a transaminase with an expanded substrate specificity (generalist enzyme) can be changed more effectively than canonical BCATs or DAATs and chose a strategy to increase the R-TA-like activity of *TaTT* by changing residues in the active site. Although our results showed that the specialization of the generalist is not straightforward, the applied strategy allowed estimating the role of individual residues in the active site in achieving different types of activity. We found that the observed *TaTT* compatibility of the high specificity toward BCAA and α-ketoglutarate and a significant activity toward R-PEA arose not only from the substitutions of residues having direct contact with the substrates but also as a result of a more subtle comprehensive structural adjustment of a functional dimer.

## Material and methods

### Expression and purification of *TaTT* variants

The *TaTT* variants were created through site-directed mutagenesis using the modified QuikChange protocol as described in [33]. Oligonucleotides used as primers for mutagenesis and mutation verification are listed in S1 Table. Eighteen cycles of PCR amplification were performed on an expression plasmid carrying a wild-type *TaTT* (WT *TaTT*) gene using the Tersus Plus PCR kit (Evrogen, Russia) and mutagenesis primers (S1 Table). Amplified fragments were treated with DpnI (Thermo Fisher Scientific, USA) restriction enzyme to eliminate the methylated matrix and then transferred to *E*.*coli* Match I сеlls. Clones carrying target mutations were identified by a colony PCR assay performed using Taq DNA polymerase and a pair of primers—a check primer specified in S1 Table and the corresponding T7 universal primer. The combination of several mutations was achieved by step-by-step point mutagenesis as described above. All selected clones were sequenced on ABI 3730xl DNA Analyzer (Applied Biosystems, USA).

The plasmid constructs were transformed into *E*. *coli* BL21(DE3)pLys cells (Stratagene, USA) for the expression of *TaTT* variants fused at the N-terminus to a His6TEV-tag. The transformed cells were grown in LB media supplemented with ampicillin (100 μg/ml) until the OD_595_ reached 0.5, then were induced with 1 mM IPTG. After overnight incubation at 30 ^o^C the cells were harvested by centrifugation at 5000 g for 20 min and resuspended in 50 mM Tris-HCl buffer, pH 8.0, containing 500 mM NaCl, 0.1% Triton, 20 mM imidazole, 1 mM PMSF, 0.2 μg/ml lysozyme and then lysed by sonication. The lysate was centrifuged at 28000 g for 30 min at 4 ^o^C. Supernatant was filtered through a 0.2 μ filter (Millipore, USA) and applied to HisTrap HP column (Cytiva, USA) equilibrated with 50 mM Tris-HCl buffer, pH 8.0, containing 500 mM NaCl, 20 mM imidazole and 0.02% (*v/v*) Triton X-100. The target proteins were eluted with a linear gradient from 40 to 500 mM imidazole in 50 mM Tris-HCl buffer, pH 8.0, containing 500 mM NaCl. The target proteins were incubated 1 h with 1 mM PLP at 30 ^o^C and transferred into storage buffer (30 mM Tris-HCl, pH 8.0, containing 100 mM NaCl, and 15 μM PLP) using PD-10 desalting columns (Cytiva). The purified proteins were concentrated up to 3–6 mg/ml with a 10 kDa cutoff centrifugal filter device (Millipore, USA) and stored in 50% glycerol at -20 ^o^C. The WT *TaTT* production was described in [15]. Briefly, the His_6_TEV-tagged *TaTT* was expressed in *E*. *coli* BL21(DE3)pLys (Stratagene). The recombinant *TaTT* was isolated using subtractive Ni-affinity chromatography and gel filtration. The fractions showing the activity were stored in 50 mM Tris-HCl buffer, pH 8.0, containing 100 mM NaCl, 15 μM PLP, and 50% glycerol at -20°C.

For crystallization, the fractions of *TaTT* mutants, after being isolated on the HisTrap HP column, were transferred into 50 mM Tris-HCl buffer, pH 8.0, supplemented with 100 mM NaCl, 1 mM EDTA, 5 mM β-mercaptoethanol, and (His)_6_-TEV protease (1 mg per 10 mg of the protein). The solution was incubated overnight at 4°C, dialyzed against 50 mM Tris-HCl buffer, pH 8.0, containing 500 mM NaCl and 20 mM imidazole, and applied to a HisTrap HP column. A (His)_6_-TEV protease and a cleaved (His)_6_-tag were absorbed on the column, and the flow-through was concentrated and applied to a Superdex 200 10/300 GL column (Cytiva) equilibrated in 15 mM Tris-HCl buffer, pH 8.0, supplemented with 50 mM NaCl and 20 μM PLP. Fractions of *TaTT* variants were concentrated up to 10–15 mg/ml and frozen at -70 ^o^C. The protein purity was analyzed by SDS-PAGE (12%). The protein concentration was determined spectrophotometrically [34].

### Enzyme activity assays

The activity assays were described in [15]. Briefly, in the indirect photometric glutamate dehydrogenase (GluDH) assay, the activities of WT *TaTT* and its variants at 0.5–1.5 μg/ml were measured in the overall transamination reaction with 5 mM L-leucine and 1 mM α-ketoglutarate in 50 mM Tris-HCl buffer, pH 8.0, supplemented with 50 mM NaCl and 60 μM PLP at 50°C. Samples (250 μl) were taken at several time points (0.5–4 min) and frozen to stop the reaction. The L-glutamate concentration was assayed in 50 mM Tris-HCl buffer, pH 9.0, supplemented with 1 mM NAD and 1.0–2.0 U GluDH (Sigma-Aldrich, USA, № G2626). One unit (U) was defined as the formation of 1 μmol L-glutamate per minute. All measurements were performed at least twice. The direct acetophenone assay was applied according to [35]. The conversion of R-PEA was determined in the reaction with 10 mM R-PEA and 10 mM pyruvate or 1 mM α-ketoglutarate in 50 mM Tris-HCl buffer, pH 9.0, supplemented with 60 μM PLP at 50°C. Acetophenone production was detected at 245 nm (an extinction coefficient of 12 mM^-1^ cm^-1^) using an Evolution 300 UV-Vis spectrophotometer equipped with a Peltier accessory (Thermo Fisher Scientific, USA). One unit (U) was defined as the formation of 1 μmol acetophenone per minute. All measurements were performed in triplicates. The data were analyzed using Origin 8.0 (Origin Lab, USA).

### Half- reaction analysis

**Fig 1:**
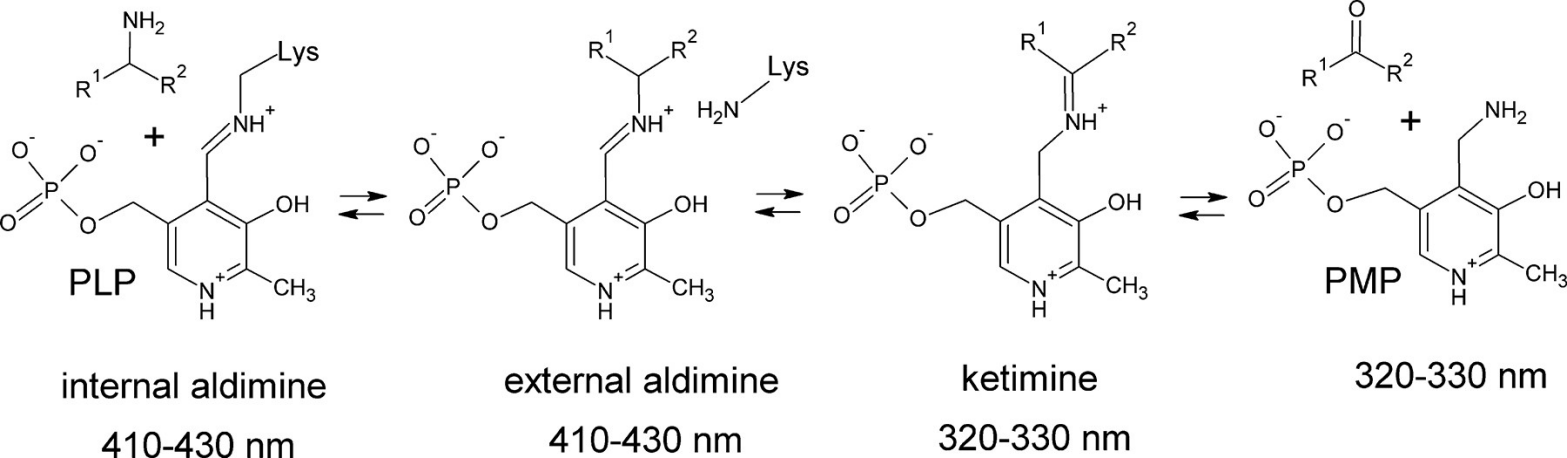
The first transaminase half-reaction.

The first transamination half-reaction is a transition of the PLP form of TA into the PMP form accompanied by the deamination of the amino donor (Fig 1). Half-reaction analysis (or pre-steady-state analysis) helps to estimate the affinity to a substrate under conditions free of the second substrate and product inhibition. The PLP forms of WT *TaTT* and its variants were obtained by incubating with the excess of both PLP and α-ketoglutarate overnight, followed by the transfer into the assay buffer using a Desalting column (Cytiva). The half-reactions of *TaTT* and its variants in PLP form (16–20 μM) with R-PEA were followed spectrophotometrically as described in [36] using an Evolution 300 UV-Vis spectrophotometer (Thermo Fisher Scientific) or SPECTROstar Omega plate reader (BMG LABTECH GmbH) in UV-transparent microtiter plates (UV-Star, Greiner Bio-One GmbH). A decrease in the aldimine concentration was measured at 410 nm with different concentrations of R-PEA (0–100 mM) in 50 mM Tris-HCl buffer, pH 8.0, at 30°C or in 50 mM CHES buffer, pH 9.0, at 40°C. The rate constants of the half-reaction were determined with nonlinear regression using Eq (1) as follows:

$$A_{t}=A_{\infty }+\Delta Aexp(-k_{obs}t),$$ (1)

where *A*_*t*_ is the absorbance at time *t*, Δ*A* is the difference between absorbance at *t* = 0 and *t* = ∞, *A*_∞_ is the final absorbance, and *k*_*obs*_ is the observed rate constant. Linear parts of the obtained kinetic curves were analyzed with the following equation: $k_{obs}=\frac{1}{A_{0}}\frac{dA}{dt}$ where A_0_ is the absorbance at t = 0. The $k_{max}^{half}$ (maximum rate constant), $K_{D}^{half}$ values (dissociation constant for the enzyme-substrate complex) and $\frac{k_{max}^{half}}{K_{D}^{half}}$ (specificity constant) for the half-reactions were determined from a plot of the observed rate constants *vs*. substrate concentration according to Eq 2:

$$k_{obs}=\frac{k_{max}^{half}[S]}{K_{D}^{half}+[S]}.$$ (2)

All measurements were performed in triplicates. The data were analyzed using Origin 8.0 software (Origin Lab, USA).

### Identification of subfamily-specific positions (SSP)

To identify SSPs (S2 Table) a multiple structure-guided sequence alignment of *TaTT* and its homologs from PLP fold type IV enzymes was constructed using the recently described Mustguseal method [37]. The PDB structure 6GKR of *TaTT* (chain A) was used as a query to run the protocol with default settings [38]. The resulting multiple alignment contained 4453 proteins and was subjected to bioinformatic analysis using Zebra2 tool to automatically classify proteins into functional families and select the corresponding specific positions [39]. The SSPs identified between the BCAT’s family and R-TA’s family were finally selected for expert inspection.

### Melting temperature determination

The apparent melting temperature (*T*_*m*_) of the *TaTT* variants was determined using the Thermofluor (also known as a Differential Scanning Fluorimetry (DSF)) assay. The samples diluted to 0.5 mg/ml in 50 mM sodium phosphate buffer, pH 8.0, containing 200 mM NaCl, 20 μM PLP and 1000× dilution of Sypro Orange dye (Sigma-Aldrich), were transferred to a 96-well PCR plates (Thermo Fischer Scientific) in the final volume 50 μl per well. Buffer mixed with Sypro dye was used as a negative control. Plates were placed in a real-time cycler (Bio-Rad C1000, with the CFX96 Real-Time accessory, USA), and the temperature was ramped up from 20 to 95°C in 1.0°C min^-1^. Relative fluorescence was measured using the FRET channel, and GraphPad Prism v8.0 (GraphPad Software Inc., USA) was used to fit the collected data a sigmoidal curve and calculate *T*_*m*_ using Boltzmann model. Assays were run in triplicate, and *T*_*m*_ was reported as the average of the three runs.

### Crystallization/data collection, structure solving, and refinement

The *TaTT* variants (mP3 and mP3O1) were crystallized by the "hanging drop" vapor diffusion method in 24-well VDX plates (Hampton Research, USA). 1.5 μl of protein was mixed with 1.5 μl of precipitant containing 100 mM HEPES, pH 7.5, 2.5–3.4 M NaCl (optimization of WT *TaTT* crystallization conditions, PDB ID 6GKR), and set up over 500 μl of precipitant in a sealed reservoir. The crystallization plates were incubated for a week at 15 ^o^C then inspected for crystal growth. The best crystals grew in 8–12 days and were rod shaped with a linear size of 200x200x100 μm. Before data collection, the crystals were briefly soaked in the mother liquor containing 25% glycerol as a cryoprotectant. They were then flash-cooled to 100 K in liquid nitrogen. The X-ray diffraction data for the *TaTT* variants were collected at the BL41XU beamline of a SPring8 synchrotron (Harima Science Garden, Japan). The data were indexed, integrated, and scaled using Dials [40]. The program Pointless [41] suggested the H32 space group for both mutants. The data collection and processing statistics are summarized in Table 1.

**Table 1:** Data collection, processing, and refinement.

*TaTT* mutants	mP3	mP3O1
Diffraction source	BL41XU, Spring8	BL41XU, Spring8
Wavelength (Å)	1.00	1.00
Temperature (K)	100	100
Detector	PILATUS	PILATUS
Crystal-to-detector distance (mm)	190.0	250.0
Rotation range per image (°)	0.5	1.0
Total rotation range (°)	100	130
Space group	H32	H32
*a*, *b*, *c* (Å)	146.50, 146.50, 143.78	146.02, 146.02, 142.12
α, β, γ (°)	90.0, 90.0, 120.0	90.0, 90.0, 120.0
Resolution range (Å)	95.13–1.75 (1.78–1.75)	94.47–1.90 (1.94–1.90)
Completeness (%)	98.6 (98.2)	98.8 (99.4)
Average redundancy	2.8 (2.8)	4.0 (4.0)
〈*I*/σ(*I*)〉	11.3 (1.0)	10.0 (0.3)
Rmeas (%) (Diederichs and Karplus 1997)	5.5 (100.1)	8.3 (440.1)
CC_1/2_ (Diederichs and Karplus 1997)	99.9 (40.1)	99.9 (12.1)
*R*_*fact*_ *(%)*	16.1	20.6
*R*_free._ *(%)*	20.1	25.5
Bonds (Å)	0.02	0.02
Angles (°)	2.03	2.22
Ramachandran plot		
Most favoured (%)	96.0	93.3
Allowed (%)	2.6	3.9
PDB entry code	7NEA	7NEB

The structures were solved by the molecular replacement method using the Molrep program [42], with the atomic coordinates of the WT *TaTT* (PDB ID: 6GKR) as a starting model. The refinement of both structures was carried out using the REFMAC5 program of the CCP4 suite [43]. In both cases, TLS was introduced during the refinement together with hydrogens in fixed positions. The electron density maps and the manual rebuilding of the model were visually inspected using the COOT interactive graphics program [44]. The resolution of the variants mP3 and mP3O1 was cut to 2.0 and 2.2 Å accordingly during the refinement to reduce the noise of the maps and achieve better R-factors. The refinement statistics for both structures are given in Table 1. In the final mP3 and mP3O1 models, an asymmetric unit contained one independent copy of the protein with 309 visible residues and covalently bound PLP molecule, 267 (117 for mP3O1) water molecules, and two (none) chloride and three (one) sodium ions from the crystallization solution. Five N-terminal and three C-terminal amino acids were invisible in the electron density map of both structures, possibly due to their high mobility.

The visual inspection of the modeled structures and figure preparation were carried out by the COOT program [44] and the PyMOL Molecular Graphics System, Version 2.4 (Schrödinger, USA). The structures were compared and superposed using the PDBeFOLD program [45]. The contacts were analyzed using the PDBePISA [46] and WHATIF software [47].

## Results

### Unveiling differences between *TaTT* and homologous BCATs and R-TAs. Selection of the sites for mutagenesis

**Fig 2:**
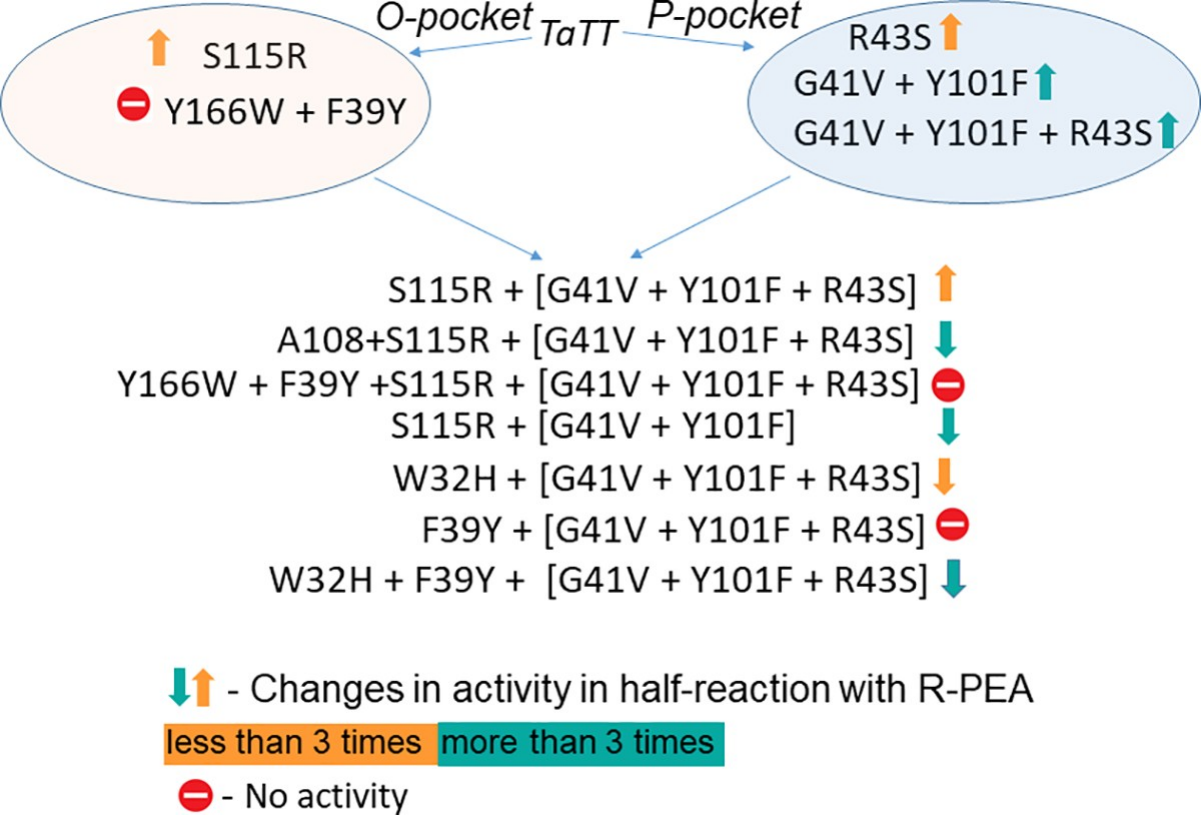
The general plan of the mutagenesis experiment.

We compared sequences and structures of *TaTT* and homologous BCATs and R-TAs and identified subfamily-specific positions (SSPs) of BCATs and R-TAs (S2 Table), which are important within these families, but differs between them. We chose the sites for mutagenesis based on the structural similarity between BCATs and R-TAs (S3 Table) and the significance of SSPs that may contribute to the selective recognition of substrates (Table 2). The general design of the mutagenesis experiment is shown in Fig 2. We considered the positions in the active site that presumably are not involved in maintaining the structural integrity of the subunits and functional dimer. Moreover, for the same reason, we did not consider the SSPs in the interior of the protein globule. The mutations were introduced in the P-pocket and O-pocket separately and in combinations as well. The P-pocket’s mutagenesis strategy entailed a *gradual substitution* of the important for BCAT-like activity residues for the residues important to R-TA-like activity. O-pocket’s mutagenesis strategy encompassed *the addition* of a key for the R-TAs residues in the O-pocket loop (S115R, insertion next to A107) and *substitutions* in the bottom of the pocket (W32H, F39Y, and Y166W). The effects of mutations were analyzed in a half-reaction with R-PEA and in the overall reactions.

**Table 2:** The amino acid content of SSPs and structural parts of the active site of *TaTT*, BCAT from *E*. *coli* and R-TA from *Aspergillus fumigatus*.

Parts of the active site:	BCAT from *E*. *coli*, (PDB ID: 1IYE)	R-TA from *A*. *fumigatus*, (PDB ID: 4CHI)	*TaTT* (PDB ID: 6GKR)
**P-pocket**:			
SSP	G39	V60	**G41**
	R40	-	**R43**
	G256	-	G259
	A259	G276	A262
Structural parts:			
αCOOH binding sites	R40 + ^256^GTAA^259^		R43 + ^259^GTHA^262^
	Y99 + R97		**Y101**
β-turn	^256^GTAA^259^	^273^TTAG^276^	^259^GTHA^262^
Interdomain loop	^125^PWGAYLGAEALE^136^	^144^PYIWVMAPENQL^155^	^130^PAVSRLEEDFS^140^
**O-pocket:**			
SSP	Y31*	H53*	S34*
	F36	Y58	**F39**
	R97	-	M103
	-	R126	S115*
	Y164	W183	**Y166**
Structural parts:			
α-COOH binding sites	-	R126*	-
left side/bottom	H27*, H30*, F36, R97, M107*, L109*, G161, Tyr164	F51*, M52*, H53*, Y58, E115, I117, L181, W183	W31*, W32*, F39, M103, L105, L163, **Y166**
O-pocket loop* (from the adjacent subunit)	^103^GDVGMGVNPPAGYS^116^	^121^GLTGVRGSKPEDLYNN^136^	^110^GNKAF**S**VVGDR^120^
**PLP coordination:**			
of phenyl group	Y164 F36	W183 Y58	**Y166 F39**
of N1-atom	E193	E212	E195
of phosphate group	R59 I220 T221 T257	R77 I237 T238 T274	R64 I222 T223 T260

Sites in *TaTT* sequence chosen for mutagenesis are shown in Bold. Residues from the adjacent subunit of the dimer are marked with an asterisk.

### The analysis of the activity of *TaTT* variants

The initial activity of *TaTT* variants in the overall reaction is summarized in Table 3 (S1 File). We estimated the changes in the *TaTT* activity toward L-leucine and α-ketoglutarate (BCAT-like activity), R-PEA and α-ketoglutarate (observed uniquely for *TaTT*), and R-PEA and pyruvate (R-TA-like activity) (S1 Fig).

**Table 3:** *TaTT* variants and their initial activities in the overall reactions between R-PEA and pyruvate, R-PEA and α-ketoglutarate, L-leucine and α-ketoglutarate.

*TaTT*	Mutations	Initial activity, U/mg			*T_m_*, °C
		R-PEA + pyruvate^a^	R-PEA + α-ketoglutarate^a^	L-Leu + α-ketoglutarate^b^	
WT	**No**	0.124 ± 0.005	0.147 ± 0.003	40 ± 5	88.0 ±0.6
		*0*.*004 ± 0*.*001*^**c**^	*0*.*002 ± 0*.*001*^**c**^	*23 ± 6*^**c**^	
mP1	**R43S**	0.095 ± 0.004	0.14 ± 0.01	0.79 ± 0.02	85.8 ± 0.4
mP2	**G41V + Y101F**	0.004 ± 0.001	0.16 ± 0.01	n.d.	84.2 ± 0.3
mP3	**R43S+ G41V + Y101F**	0.004 ± 0.001	0.018 ± 0.006	n.d.	86.2 ± 0.2
		*0*.*008 ± 0*.*001*^**c**^	*0*.*008 ± 0*.*001*^**c**^		
mO1	**S115R**	0.112 ± 0.005	0.064 ± 0.003	1.8 ± 0.4	n.m.
mO2	**Y166W + F39Y**	n.d.	n.d.	n.d.	83.9 ±0.2
mP2O1	**G41V + Y101F + S115R**	0.003 ± 0.001	0.004 ± 0.001	n.d.	n.m.
mP3O1	**R43S + G41V + Y101F + S115R**	0.003 ± 0.001	0.024 ± 0.002	n.d.	82.9 ± 0.4
		*0*.*012 ± 0*.*008*^**c**^	*0*.*066 ± 0*.*003*^**c**^		
mP3O3	**R43S + G41V + Y101F + S115R + A108**	*0*.*007 ± 0*.*002*^**c**^	n.d.	n.m.	n.m.
mP3O4	**R43S + G41V + Y101F + S115R + Y166W + F39Y**	n.d.	n.d.	n.d.	n.m.
mP3O5	**R43S + G41V + Y101F + W32H**	n.m.	0.008 ± 0.001	n.d.	n.m.
mP3O6	**R43S + G41V + Y101F + F39Y**	n.d.	n.d.	n.d.	n.m.
mP3O7	**R43S + G41V + Y101F + F39Y + W32H**	n.m.	0.003 ± 0.001	n.d.	n.m.

Variants containing changes in P-pocket are named mP1-mP3; variants containing changes in O-pocket are named mO1-mO2; variants including changes in both pockets are named mPxOx accordingly. Apparent melting temperature is shown as well.

^a^Initial activity measured at pH 9.0 using the acetophenone assay

^b^Initial activity measured at pH 8.0 using the GDH assay

^c^The reaction was carried out at pH 7.0 (50 mM Tris-HCl, 50°C)

n.m. = not measured

n.d. = not detected. The detection limit of the acetophenone assay is 0.0001 U/mg. The detection limit of the GluDH assay is 0.08 U/mg.

All mutations were unfavorable for any types of *TaTT* activity in the overall reaction, with BCAT-like activity being the most susceptible to the changes. Only variants mP1and mP2 were comparable to WT *TaTT* in the reaction R-PEA + α-ketoglutarate. We observed an increase in R-TA-like activity at pH 7; interestingly, mP3O1 (Table 3) was more active toward R-PEA at pH 7.0 than at pH 9.0. The introduction of double mutations (Y166W + F39Y) was fatal for all types of activity (see mO4 and mP3O4 in Tables 3 and 4). It worth noting that according to the *T*_*m*_ values (S2 File), no mutations induced denaturation of the *TaTT* globule. In other words, the chosen mutagenesis positions did not affect the structural integrity of *TaTT*.

**Table 4:** The activity of PLP form of *TaTT* variants in half-reaction with R-PEA.

*TaTT*	Mutations	pH 8.0, Tris-HCl buffer, 30°C			pH 9.0, CHES buffer, 40°C		
		*k*_max_,	*K*_*D*_,	k_max_/*K*_*D*_,	*k*_max_,	*K*_*D*_,	*k*_max_/*K*_*D*_,
		s^-1^	mM	s^-1^M^-1^	s^-1^	mM	s^-1^M^-1^
WT	**No**	0.030 ± 0.001	82 ± 6	0.36 ± 0.03	0.073 ± 0.004	81 ± 8	0.9 ± 0.1
mP1	**R43S**	0.030 ± 0.002	110 ± 13	0.27 ± 0.04	n.m.	n.m.	n.m.
mP2	**G41V + Y101F**	No saturation		2.1 ± 0.5	0.28 ± 0.02	13 ± 2	20 ± 3
mP3	**R43S+ G41V + Y101F**	No saturation		10.0 ± 0.5	0.15 ± 0.01	2.6 ± 0.4	60 ± 10
mO1	**S115R**	0.050 ± 0.004	48 ± 8	1.0 ± 0.2	0.011 ± 0.001	3.3 ± 1.2	3.3 ± 1.2
mO2	**Y166W + F39Y**	n.d.			n.d.		
mP2O1	**G41V + Y101F + S115R**	No saturation		5.7 ± 0.2	n.m.	n.m.	n.m.
mP3O1	**R43S + G41V + Y101F + S115R**	No saturation		35 ± 1	0.13 ± 0.01	1.5 ± 0.5	90 ± 30
mP3O3	**R43S + G41V + Y101F + S115R + A108**	0.9 ± 0.2	101 ± 22	9 ± 3	n.m.	n.m.	n.m.
mP3O4	**R43S + G41V + Y101F + Y166W + F39Y**	n.d.			n.d.		
mP3O5	**R43S + G41V + Y101F + W32H**	No saturation		6.5 ± 0.8	n.m.	n.m.	n.m.
mP3O6	**R43S + G41V + Y101F + F39Y**	n.d.			n.d.		
mP3O7	**R43S + G41V + Y101F + F39Y + W32H**	0.011 ± 0.001	44 ± 8	0.25 ± 0.05	n.m.	n.m.	n.m.

Variants containing changes in P-pocket are named mP1-mP3, variants containing changes in O-pocket are named mO1-mO4; variants with changes in both pockets are named mPxOx accordingly.

n.m. = not measured; n.d. = not detected

To detail the effects of mutations on the R-TA-like activity, we focused on half-reactions with R-PEA at pH 8.0 and 9.0 (pH 9.0 was optimal for *TaTT* in reactions with R-PEA [15], while pH 8.0 was optimal for *TaTT* in reactions with L-amino acids [15]). The parameters for the half-reactions of variants are collected in Table 4 (S3 File). Positive effects were observed at both pHs; a more effective binding of R-PEA and saturation were observed predominantly at pH 9.0. The variant mP3O1 showed the highest specificity for R-PEA: three mutations in the P-pocket together with a mutation S115R in the O-pocket loop improved the productive binding of R-PEA as well as the effectiveness of the amino group transfer. It is noteworthy that the achieved effect was not a sum of the effects induced by the separate mutations in the P-pocket (mP1 + mP2) and O-pocket (mO1); rather, it was the cumulative effect of the four mutations (G41V + Y101F + R43S + S115R).

Any other changes in the O-pocket combining with the three mutations in the P-pocket were unsuccessful. The double mutation (Y166W + F39Y) was fatal for the *TaTT* activity. Mutations W32H and F39Y (in mP3O5, mP3O6, and mPeO7) destroyed the *TaTT* activity toward R-PEA as well.

The half-reaction analysis of the variant mP3O1 at pH 7.0 (50 mM Tris-HCl, 40°C) showed no saturation; the specificity constant for R-PEA was (2.6 ± 0.2) s^-1^M^-1^, which is 30 times lower than that at pH 9.0. For WT *TaTT*, the half-reaction analysis at pH 7.0 showed no saturation as well, with the specificity constant for R-PEA being (0.009 ± 0.001) s^-1^M^-1^. Apparently, the observed increase in the activity of mP3O1 in the overall reactions with R-PEA at pH 7.0 (Table 3) indicated a better binding of the second substrate at a neutral pH.

### Structural analysis of functional dimers of mP3 and mP3O1

**Fig 3:**
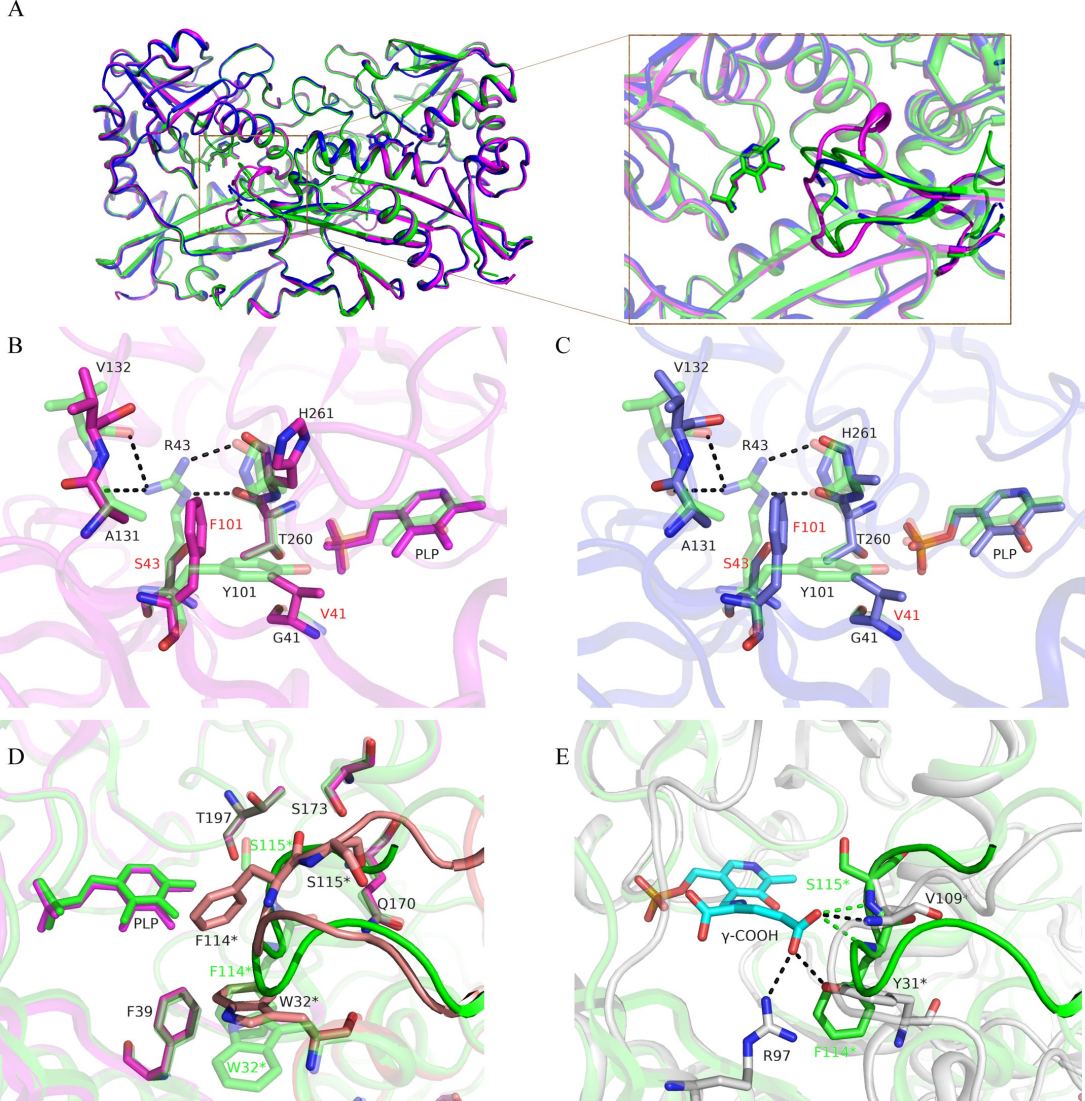
Structural comparison of WT *TaTT* and its variants. (A) Superposition of WT *TaTT* (green), mP3 (magenta) and mP3O1 (blue) dimers. PLP molecules are shown as sticks. Insert is a zoomed view on a region of the O-pocket loop with the major changes between the structures (the other part of molecules are shown transparently for clarity). (B) Active center superposition between WT *TaTT* (green, transparent) and mP3 (pink). Hydrogen bonds are shown as dashed lines. Mutated in mP3 residues are labeled in red. (C) Active center superposition between WT *TaTT* (green, transparent) and mP3O1 (blue). Label coding is similar to panel B. (D) Comparison of the O-pocket loop regions in WT *TaTT* (green) and mP3 (pink). O-pocket loop in mP3 is colored in coral. The rest of the molecules are transparent. Important O-pocket loop residues of the WT *TaTT* are labeled in green. Residues from another subunit of the dimer are marked with an asterisk. (E) Comparison of α-ketoglutarate γ-COOH group binding in *E*.*coli* BCAT (white, PDB ID 1IYE) and WT *TaTT* (green). α-ketoglutarate covalently bound to PLP molecule is shown with cyan. Corresponding hydrogen bonds are shown as dashed lines.

To shed light on the structural basis of the abovementioned mutation effects, we obtained the structures of two variants—mP3 and mP3O1. The superposition of the subunits showed that the backbones of both variants and the WT *TaTT* are similar: pairwise RMSD between the Cα atoms of subunits did not exceed 0.3 (S4 Table). Major differences were found in the conformation of the O-pocket loop and the interactions between the residues of this loop and residues forming the P-pocket and the interdomain loop (Fig 3A).

In WT *TaTT*, the side chain of R43 forms hydrogen bonds with the backbone atoms of the H261 and T260 residues from the β-turn ^259^GTHA^262^ and could simultaneously form hydrogen bonds with A131 and V132 residues from the interdomain loop ^130^PAVSRLEEDFS^140^ (Fig 3B). In other words, R43 arranges both the α-COOH binding site in the P-pocket via the hydrogen bonding with the backbone atoms of the β-turn and the active site entrance by fixing the interdomain loop. Mutation R43S in both variants led to a shift of the β-turn and the interdomain loop toward the active site: about 0.6 Å between the corresponding Cα atoms of H261 and V132 (Fig 3B and 3C). In WT *TaTT*, R43 was involved in cation-π interaction with H261. Substitution of a side chain of R43 induced the flexibility of the H261 side chain in both variants, which is reflected by the high B-factor (mP3) or even the absence of the electron density for the side chain (mP3O1) (Fig 3C). When Y101 was substituted for phenylalanine, the side chain of the latter oriented almost 180° away from the P-pocket in both variants and accepted the conformation induced by both the R43S and G41V changes (Fig 3B and 3C). The former created a void for phenyl moiety, occupied in WT *TaTT* by the R43 side chain, while the latter expelled the F101 side chain into this void.

There are large conformation changes in the O-pocket loop in the mP3 and mP3O1 variants (Fig 3A); moreover, in mP3O1, this loop is partially disordered (residues 113–117 have no clear electron density). Compared to WT *TaTT*, the mP3 O-pocket loop lacks some stabilizing hydrogen bonds—between the backbone oxygen of S115* and the side chains of T197 and S173 and between the backbone oxygen of F114* and the side chain of Q170. These disturbances induced a shift of the loop, and thereby, the side chain of F114* shifted toward the cofactor and partially blocked the entrance into the active site (Fig 3D). In WT *TaTT*, F114* is located opposite F39 and forms the stacking interactions with W32*, while in mP3, the side chain of W32* occupies the WT position of F114*. Interestingly, the W32* side chain orientation and the conformation of the O-pocket loop appear mutually related. The S115R mutation in the O-pocket loop induced its disordering and increased the W32* side chain flexibility. In mP3O1 (mP3 + S115R), W32* has two conformations: one is similar to that in mP3, and the other is unique and occupies the WT position of the O-pocket loop main chain.

In sum, the substitutions induced a rearrangement in the active site, including an enhancement of hydrophobicity and an increase in the O-pocket loop and P-pocket β-turn mobility.

## Discussion

The development of effective strategies to design biocatalysts for organic synthesis requires reliable algorithms based on *sequence-structure-function* relationships [48, 49]. Such an experience is needed for the application of transaminases for the (*R*)-selective amination as well. In 2010, Höhne et al. [14] described two specificity-determining sequence motifs, which differ between three canonical families of TA of PLP fold type IV (S5 Table). The observed *sequence-function* relationships encouraged the researchers to change the specificity of TAs by replacing key residues within similar structural scaffolds. Voss et al. [32] succeeded at changing the activity of DAAT from *B*. *subtilis* to R-TA-like activity. The mutated DAAT showed 0.33 U/mg activity in converting R-PEA but lost its native activity toward D-amino acids (the conversion rate of natural R-TAs lies in the range of 0.1–10 U/mg [14, 50]). The same experiment with BCAT from *E*. *coli* was unsuccessful [32]. In both experiments, the authors worked with specialist enzymes (monofunctional). We tried to modify the specificity of the generalist enzyme *TaTT* that exhibits BCAT-like activity with BCAA/L-phenylalanine and α-ketoglutarate/pyruvate, producing α-keto acids and L-glutamate/L-alanine (*Vmax* is 180 U/mg and 46 U/mg, respectively) as well as R-TA-like activity with R-PEA and α-ketoglutarate/pyruvate, producing acetophenone and L-glutamate/L-alanine (*Vmax* is 0.33 U/mg). Earlier, we suggested that the expanded substrate specificity of *TaTT* resulted from the changes in the specificity-determining sequence motifs (S5 Table). These changes appeared to diminish a rigid BCAT-like organization of the P-pocket and increase the O-pocket’s hydrophobicity (S3 Fig), thus favoring the binding of aromatic L-amino acids and the phenyl and methyl moiety of R-PEA as well. As shown in the current work, further modifications imitating the active site of R-TAs not only destroyed the BCAT-like activity but failed to improve the R-TA-like activity of *TaTT*. Meanwhile, the obtained data revealed the role of the individual residues within the *TaTT* active site in expanding substrate specificity.

In canonical BCATs, P-pocket contains two α-COOH group binding sites: one is formed by the phenyl group of the tyrosine residue from the βY-strand (Y101 in *TaTT*), polarized by the neighboring conserved arginine residue, the other is formed by the backbone atoms of the b-turn polarized by the other conserved arginine residue from the βX-strand (R43 in *TaTT*) [19, 28, 29] (S2 Fig). The mutation R43S induced a dramatic decrease in BCAT-like activity of *TaTT*, and no severe change in R-TA-like activity was observed (Table 3). The double substitution G41V + Y101F (variant mP2) also destroyed the BCAT-like activity and induced a dramatic decrease in *TaTT* activity in the R-PEA + pyruvate reaction. However, it did not influence the *TaTT* activity in the R-PEA + α-ketoglutarate reaction. These observations underpinned the significance of the P-pocket organization for BCAT-like activity and indicated the multipoint binding of α-ketoglutarate. Interestingly, Y101 is nonpolarized in *TaTT*, contrary to canonical BCATs. However, Y101 appeared to keep the proper coordination of the α-COOH group of pyruvate and L-leucine, even in a nonpolarized state. The triple mutation G41V + Y101F + R43S (in mP3) destroyed the binding of the α-COOH group in *TaTT* and led to the loss of both BCAT-like and R-TA-like activities. However, triple mutation improved the affinity to R-PEA more than 60 times.

The mP3O1 variant, harboring a triple mutation in the P-pocket and the mutation S115R in the O-pocket loop, showed the highest affinity to R-PEA. A single S115R mutation improved R-PEA binding in WT *TaTT* as well. The improvement can be attributed to the incorporation of a positive charge in the O-pocket, which appears to adjust R-PEA coordination by fixing the aromatic moiety away from the charged side chain of R115. These changes imitated the key residues in the active site of natural R-TAs and appeared to favor the R-PEA binding. Further substituting the unique *TaTT* residues W32H and F39Y for R-TA ones at the bottom of the O-pocket (in mP3O5, mP3O6, and mPeO7) decreased the *TaTT* affinity to R-PEA. Double substitution Y166W + F39Y induced the loss of both *TaTT* activities. The structural and SSP analysis of BCATs showed that the tyrosine residue (Y166 in *TaTT*) is coupled with phenylalanine residue (F39 in *TaTT*), and this couple is essential for function and PLP-coordination; the latter in *TaTT* occur similarly to canonical BCATs (S4A Fig) [15, 19, 26, 27]. Tyrosine is suggested to move during the catalytic process [15, 19]. In R-TAs, another couple of residues, tryptophan and tyrosine (W183 + Y58 in R-TA from *A*.*fumigatus*, (S4B Fig), seems to coordinate with the phenyl group of the PLP through the water molecule; however, their contribution to the catalysis is unclear [18]. Whereas the coordination of phosphate group of PLP is universal among TAs of PLP-fold type IV, the interactions with the phenyl group of PLP appears to be specific among canonical families and are consistent with the O-pocket organization and ultimately substrate specificity. The *TaTT* inactivation due to the double substitution Y166W + F39Y (mP3O4 and mO4) underpinned the specificity of PLP coordination. At the structural level, the inactivation of *TaTT* seems to result from the reduced mobility of the introduced tryptophan and tyrosine residues compared to their analogs in native R-TAs. In R-TAs, the tryptophan residue (S3 Fig) is located at the loop next to the α-helix, while the similar position in *TaTT* is on the α-helix. An ordered arrangement of the α-helix presumably does not allow the tryptophan residue to take the proper position in the variants. The insertion A108 in the O-pocket loop in mP3O3 aimed to elongate the loop as in canonical R-TAs [13, 25, 51] failed to improve the R-TA-like activity of *TaTT* as well.

Effective α-ketoglutarate binding is a prominent feature of *TaTT* catalysis: α-ketoglutarate is the best substrate, and its amination does not limit the overall reaction rate [13, 15]. The binding of α-ketoglutarate in *TaTT* generally reproduces the binding of α-ketoglutarate in typical BCATs [13, 15] except the coordination of a γ-COOH group. In typical BCATs, the γ-COOH group is coordinated by tyrosine residue from the βX-strand, arginine residue from βY-strand and the backbone NH group of leucine(valine) residue from the O-pocket loop (PDB ID: 5E25, 1IYE). In *TaTT*, the γ-COOH group appears to coordinate solely with the backbone NH groups of F114* and S115* on the O-pocket loop (Fig 3E). The triple mutation in the P-pocket induced a significant shift of the loop accompanied by a rearrangement of hydrogen bonding between the O-pocket loop and the structural elements forming the P-pocket. Consequently, the α-ketoglutarate binding sites are disturbed in mP3. Moreover, the side chain of F114* is placed in a potential position of the γ-COOH group and can prevent proper binding of α-ketoglutarate (Fig 3D and 3E). Notably, the single S115R mutation decreased the activity of *TaTT* in the R-PEA+ α-ketoglutarate and L-leucine + α-ketoglutarate reactions. The change in the O-pocket loop seems to make the α-ketoglutarate amination a rate-limiting step even with such a "slow" substrate like R-PEA. These results support the importance of the O-pocket loop for the productive binding of α-ketoglutarate and the multipoint binding of α-ketoglutarate. However, a single mutation S115R does not influence the pyruvate amination, presumably due to the lack of the pyruvate-binding sites in the O-pocket. As an imitation of the R-TAs’ pyruvate-binding site, the introduction of S115R in mP3 was unsuccessful: the productive binding of pyruvate in the proD-position (through the coordination of the α-COOH group with the side chain of R115 in the O-pocket loop) was not achieved. The HPLC analysis of the products of transamination between R-PEA and pyruvate catalyzed by mP3O1 revealed only L-alanine (S5 Fig).

In summary, the idea to specialize the *TaTT* by introducing residues from the active site of R-TAs into the active site failed. Although we improved the *TaTT* specificity toward R-PEA, we disturbed the reaction mechanism in total. We assume that the nature of the expanded substrate specificity of *TaTT* is more complex than a combination of residues specific to BCATs and R-TAs in one active site. In other words, *TaTT* is not a "transition form" between BCATs and R-TAs but a unique enzyme. We suggest that the activity of *TaTT* toward R-PEA and the high catalytic efficiency for α-ketoglutarate and BCAA are achieved through several factors in the active site, including a unique combination of hydrophobic residues, the relaxation of some hydrogen bonds, and a peculiar O-pocket loop arrangement.

## Conclusions

PLP-dependent TAs of fold type IV are informative objects in studying *sequence-structure-function* relationships due to the high structural similarity, which helps differentiate the position between "important for structure" and "important for function." In the current research, we studied the effects of mutations in the "important for function" positions on the transaminase from *T*. *terrenum* activity. *TaTT* is distinguished by an expanded substrate specificity: it can catalyze the amino group transfer between BCAAs and α-ketoglutarate, producing α-keto acids and L-glutamate (benchmark reactions for BCATs), and between R-PEA and α-ketoglutarate producing ketone and L-glutamate (R-TA-like activity). The latter is a unique reaction because canonical R-TAs are inactive with α-ketoglutarate and catalyze the amino group transfer between R-PEA and pyruvate, producing acetophenone and D-alanine. The specificity for R-PEA arises without sacrificing the specificity for L-Leu and α-ketoglutarate and in consensus with it: *TaTT* demonstrates one of the highest rates of α-ketoglutarate conversion. Incorporating key residues of canonical R-TAs in the *TaTT* active site, we succeeded in enhancing the affinity to R-PEA; however, we destroyed the activity in the overall transamination reactions due to the lack of a proper binding of substrates with the α-COOH group. We found that the active site of *TaTT* is characterized by the relaxation of some conserved for BCATs interactions, which favored the specificity for R-PEA and enhanced the specificity for α-ketoglutarate.

## Supporting information

S1 Fig Benchmark reactions catalyzed by members PLP fold type IV superfamily. Unique reactions catalyzed by *TaTT*.
(PDF)

S2 Fig The active site of PLP-dependent TAs of fold type IV. (PDF)

S3 Fig Schematic representation of the active site of *TaTT*. (PDF)

S4 Fig Coordination of the phenyl group of PLP. (PDF)

S5 Fig Analysis of the products of the overall transamination reaction, catalyzed by mP3O1. (PDF)

S1 Table List of primer sequences for the *TaTT* mutagenesis. (PDF)

S2 Table Subfamily-specific position among BCAT and R-TA families of PLP fold type IV. (PDF)

S3 Table Superposition of the *TaTT* subunit with the closest homologs. (PDF)

S4 Table RMSD (Å) between Cα atoms of subunits of WT *TaTT* and its variants. (PDF)

S5 Table Specificity determining sequence motifs of TaTT and TAs from the families of PLP fold type IV. (PDF)

S1 File Determination of the initial activity of *TaTT* and its variants in the overall reaction. (PDF)

S2 File Determination of the melting temperature of *TaTT* and its variants. (PDF)

S3 File Analysis of half-reactions of *TaTT* and its variants. (PDF)
