# Supplementary material for: Probing the role of the residues in the active site of the transaminase from Thermobaculum terrenum
Source: PLoS One. 2021 Jul 29;16(7):e0255098. doi: 10.1371/journal.pone.0255098 (PMC8320979; doi:10.1371/journal.pone.0255098)
Supplement: S1 Fig — Unique reactions catalyzed by TaTT. (PDF) [file pone.0255098.s001.pdf]

Figure S1. Benchmark reactions catalyzed by members of PLP fold type IV superfamily. Unique reactions catalyzed by *TaTT*.

Benchmark reaction of DAATs

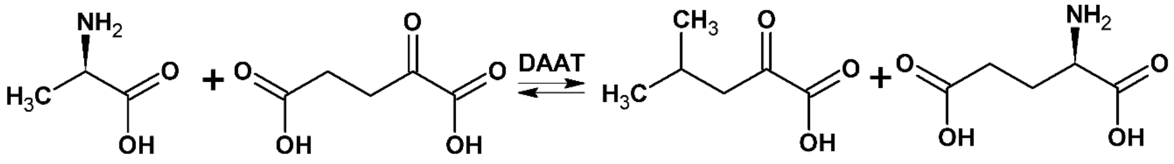

D-Ala α-ketoglutarate 4-methyl-2-oxovalerate D-Glu

Benchmark reaction of BCATs

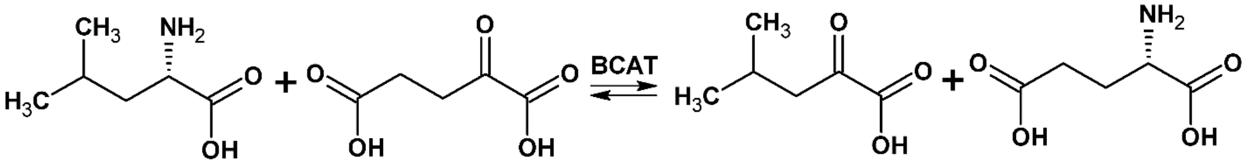

L-Leu α-ketoglutarate 4-methyl-2-oxovalerate L-Glu

Benchmark reaction of R-TAs

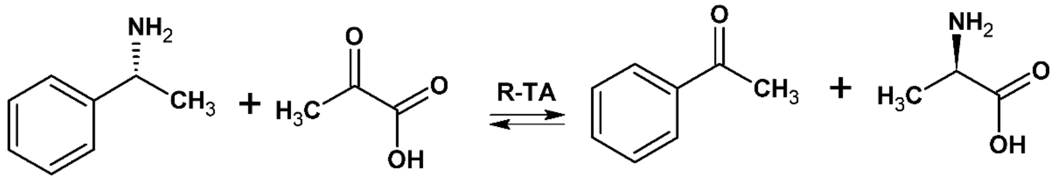

R-PEA pyruvate acetophenone D-Ala

Unique reactions catalyzed by *TaTT*

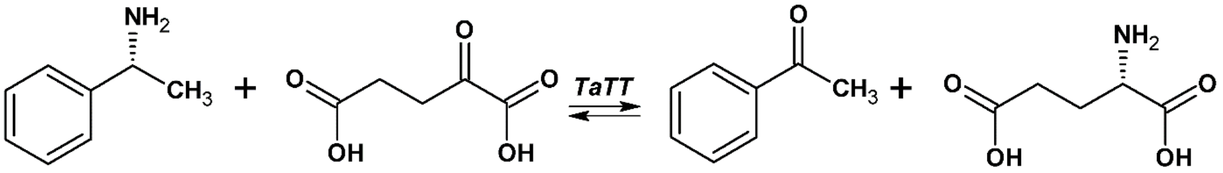

R-PEA α-ketoglutarate acetophenone L-Glu

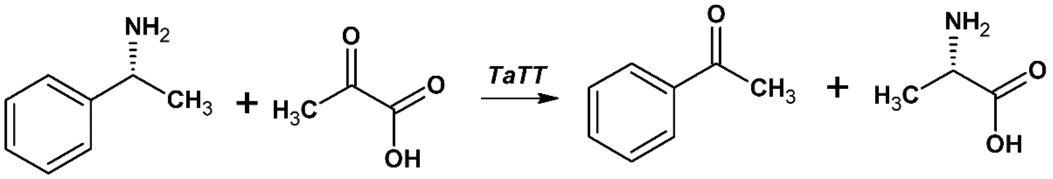

R-PEA pyruvate acetophenone L-Ala
