# Supplementary material for: Probing the role of the residues in the active site of the transaminase from Thermobaculum terrenum
Source: PLoS One. 2021 Jul 29;16(7):e0255098. doi: 10.1371/journal.pone.0255098 (PMC8320979; doi:10.1371/journal.pone.0255098)

Figure S2. The active site of PLP-dependent TAs of fold type IV is exemplified by a canonical BCAT from *E. coli* (PDB ID 1I1L). One subunit of the dimer is gray. The other is colored: large and small domains are in yellow and green, respectively. One side and the bottom of both active site pockets are formed by the residues from the βX- and βY-strands (cyan) of one subunit of the active dimer. The interdomain loop (orange) and β-turn (black) of the large domain of this subunit confine the P-pocket from the other sides. The O-pocket loop of the second subunit (red) of the dimer complements a side and the lid of the O-pocket together with two α-helices from both subunits.

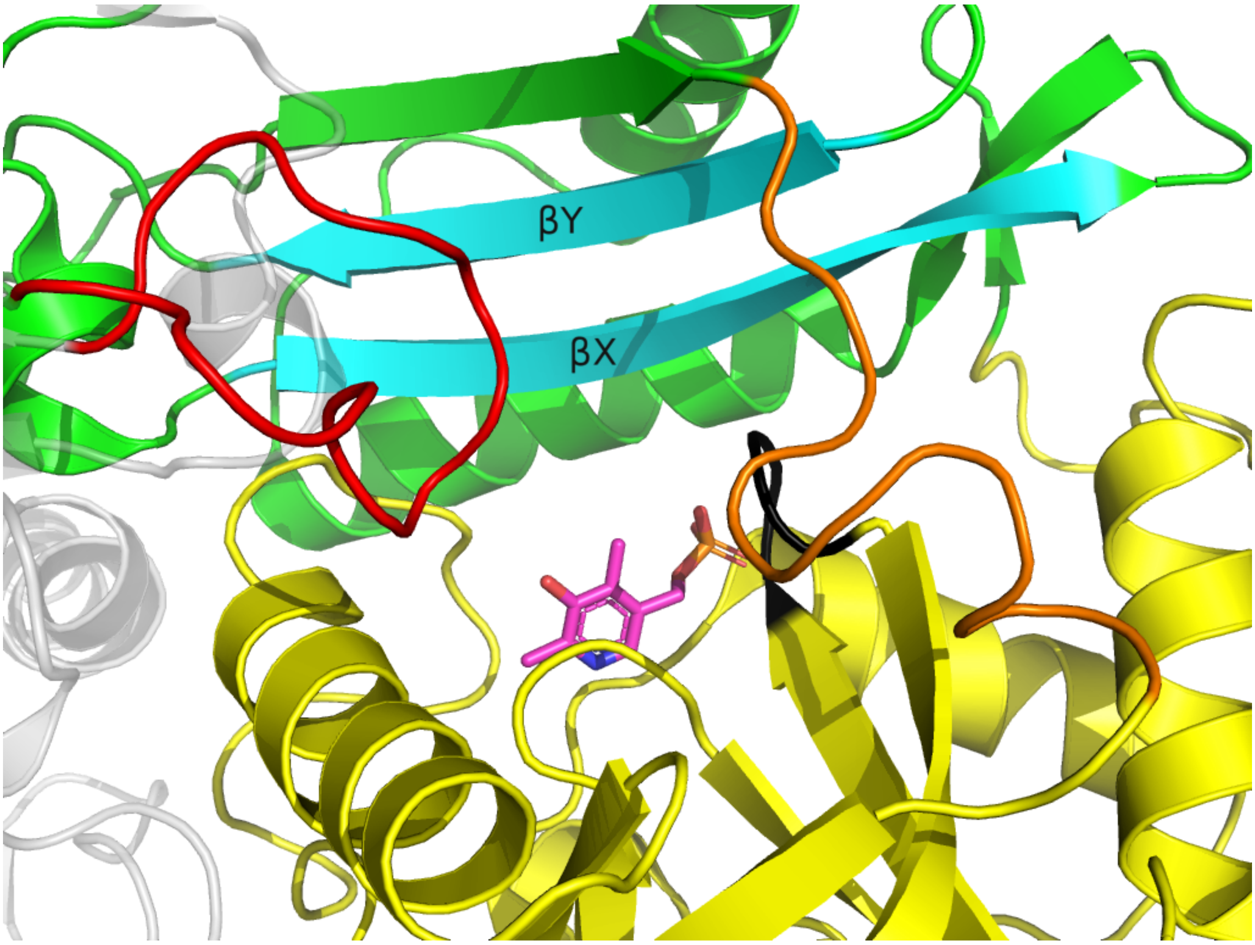

Supplement: S2 Fig — (PDF) [file pone.0255098.s002.pdf]
