# Supplementary material for: Probing the role of the residues in the active site of the transaminase from Thermobaculum terrenum
Source: PLoS One. 2021 Jul 29;16(7):e0255098. doi: 10.1371/journal.pone.0255098 (PMC8320979; doi:10.1371/journal.pone.0255098)

Figure S3. Schematic representation of the active site of *TaTT*. The inner surface of the active site is shown as a semitransparent surface. PLP molecule covalently bound to catalytic lysine is pink. Important residues of the P- and O-pockets are shown in blue and cyan sticks, respectively, and labeled. Protein fold is shown as a yellow cartoon for clarity.

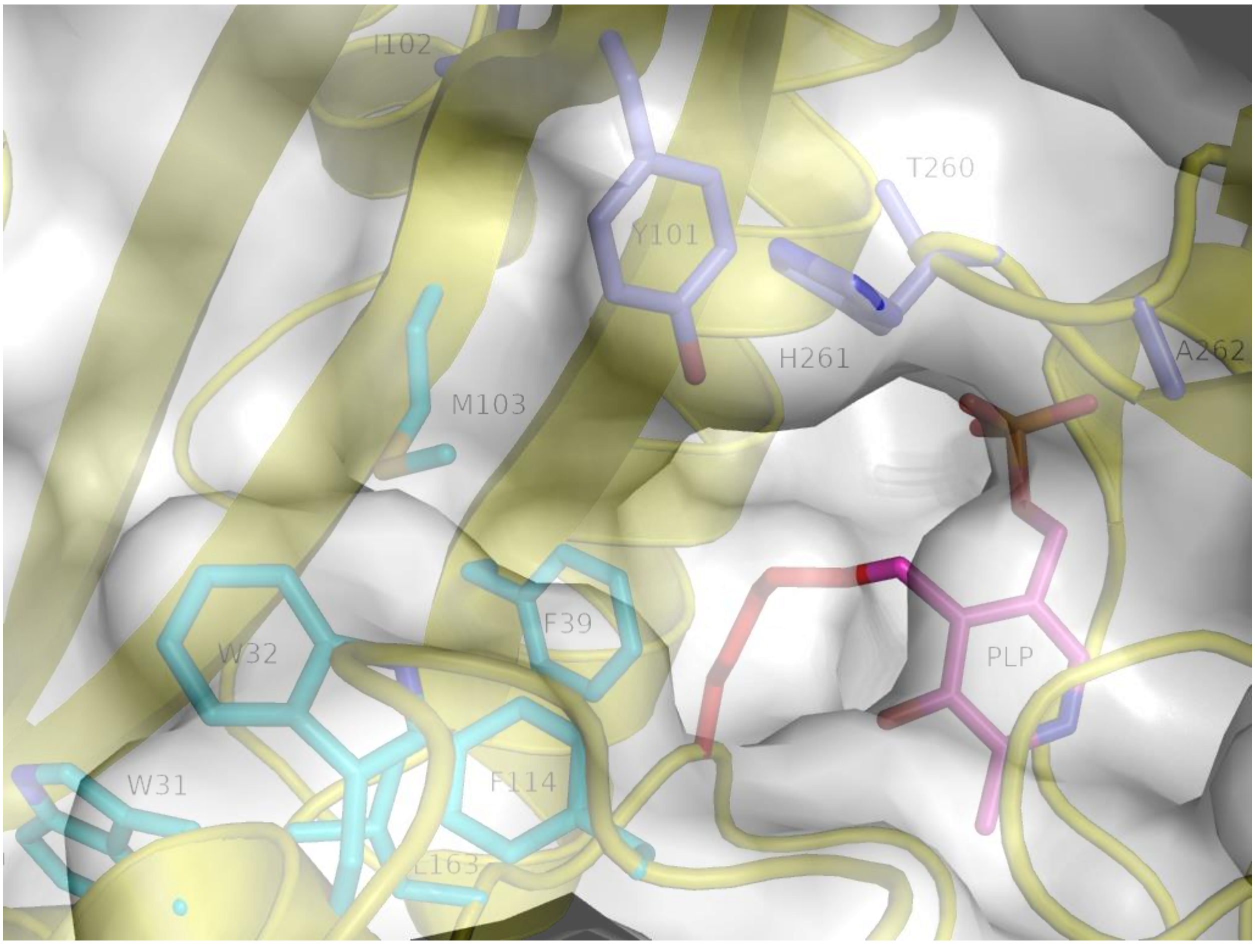

Supplement: S3 Fig — (PDF) [file pone.0255098.s003.pdf]
