# Supplementary material for: Probing the role of the residues in the active site of the transaminase from Thermobaculum terrenum
Source: PLoS One. 2021 Jul 29;16(7):e0255098. doi: 10.1371/journal.pone.0255098 (PMC8320979; doi:10.1371/journal.pone.0255098)

Figure S4. Coordination of the phenyl group of PLP. A) Position of the couple Y166 + F39 in *TaTT* (pink, PDB ID: 6GKR) and BCAT from *E. coli* (blue, PDB ID: 1IYK); B) Position of the couple W183 + Y58 in R-TA from *Nectria haematococca* (PDB ID: 4CMD).

A

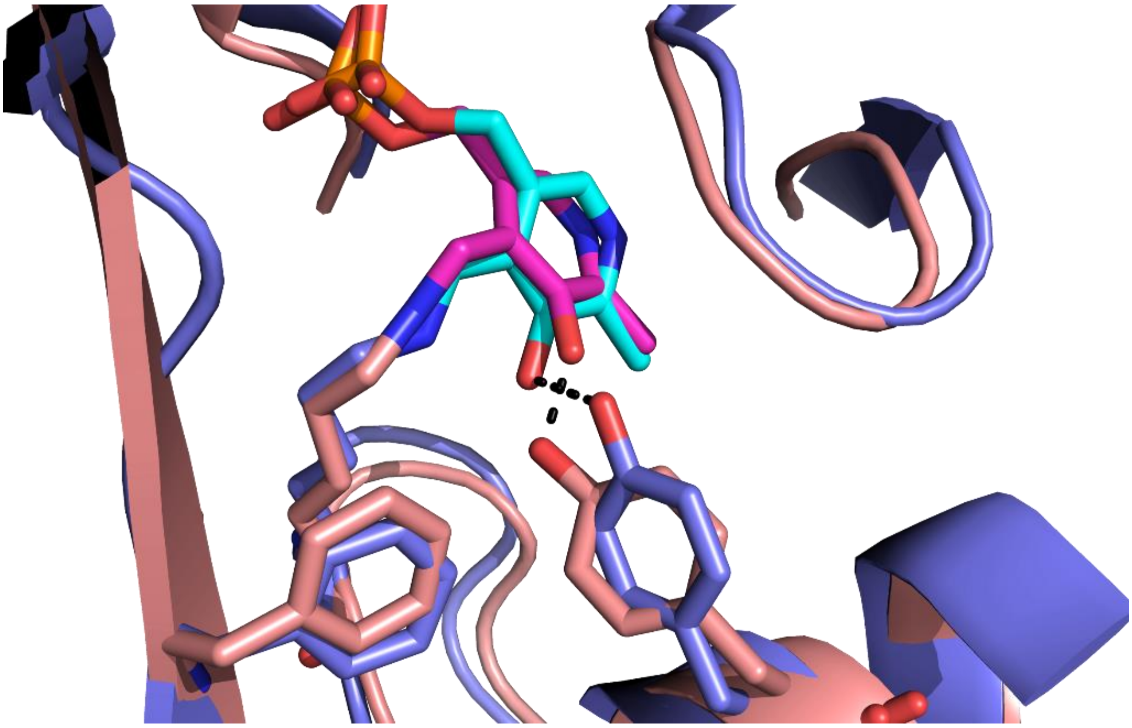

B

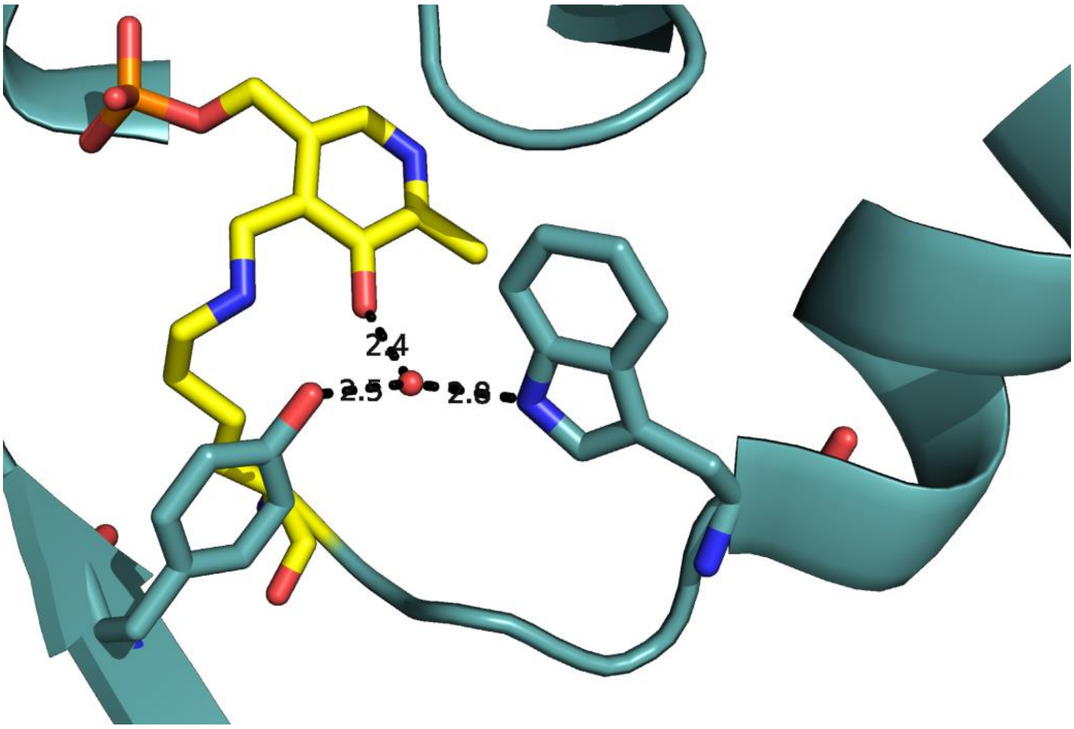

Supplement: S4 Fig — (PDF) [file pone.0255098.s004.pdf]
