# Supplementary material for: Probing the role of the residues in the active site of the transaminase from Thermobaculum terrenum
Source: PLoS One. 2021 Jul 29;16(7):e0255098. doi: 10.1371/journal.pone.0255098 (PMC8320979; doi:10.1371/journal.pone.0255098)
Supplement: S5 Fig — (PDF) [file pone.0255098.s005.pdf]

S5 Figure. Analysis of the products of the overall transamination reaction, catalyzed by mP3O1.

Reaction: R-PEA + pyruvate ↔acetophenone + Ala (D- or L-)

Reaction conditions: 50 mM Tris-HCl, pH 7.0, containing 60 μM PLP, 15 mM R-PEA and 15 mM pyruvate at 50 °C..

Determination of the products: The aliquots were taken from the reaction mixture after 7 h and 14 h. The protein was removed by centrifugation using Amicon 10 devices (Millipore, USA). The enantiomeric excesses were determined (within the limits of detection) by chromatographic analysis of the supernatants using a CROWNPAK_ CR(+) column (150 x 4 mm x 5 μm; Daicel, Illkirch, France) with UV detection at 200 nm. +**25 °C**: A) mixture of 1 mM D-Ala and 1 mM L-Ala in 50 mM Tris-HCl, containing 60 μM PLP and 5 mM pyruvate; B) reaction mixture. **+4 °C**: C) mixture of 1 mM D-Ala and 1 mM L-Ala in 50 mM Tris-HCl, containing 60 μM PLP and 5 mM pyruvate; D) reaction mixture .

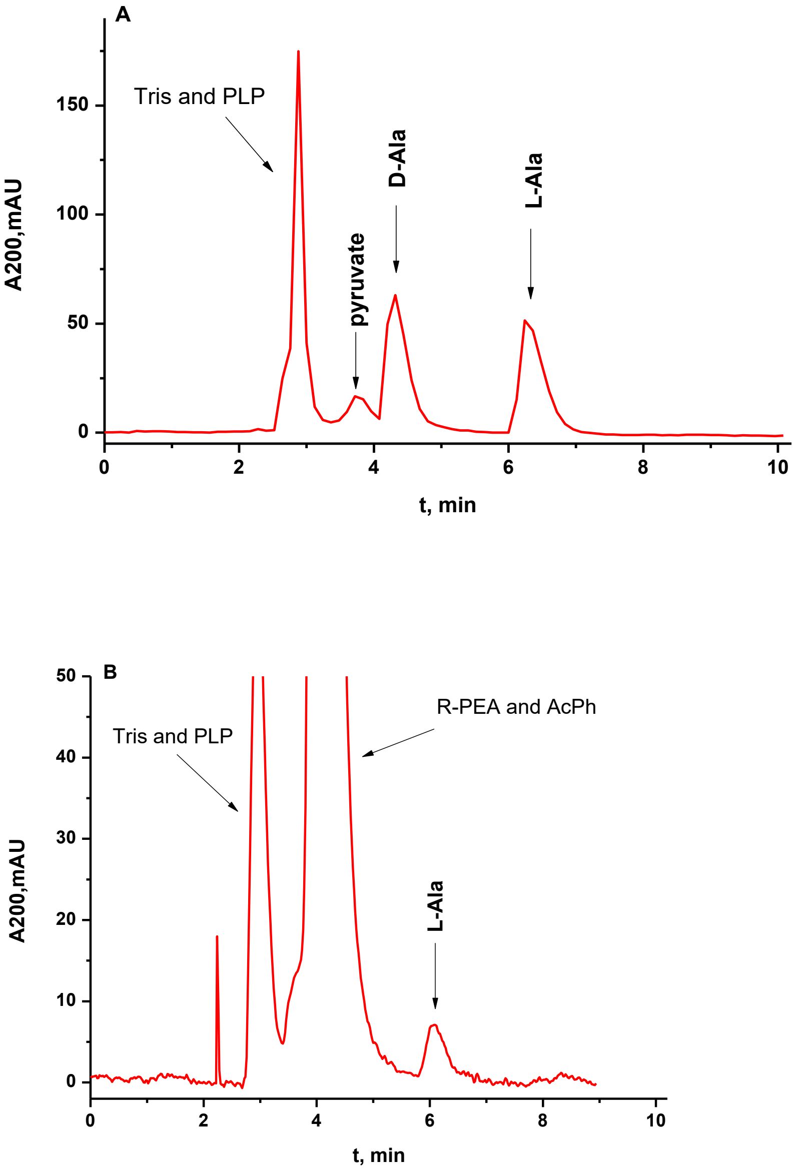

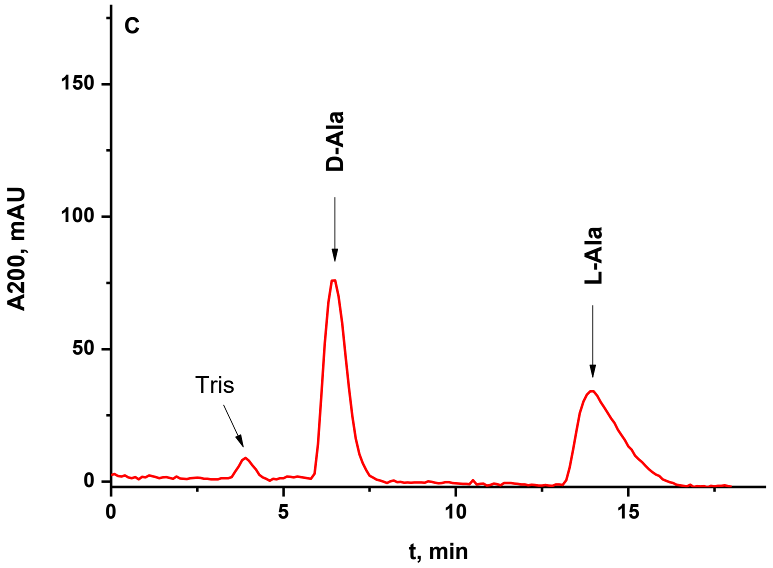
C
A200, mAU
150
100
50
0
D-Ala
L-Ala
Tris
0
5
10
15
t, min

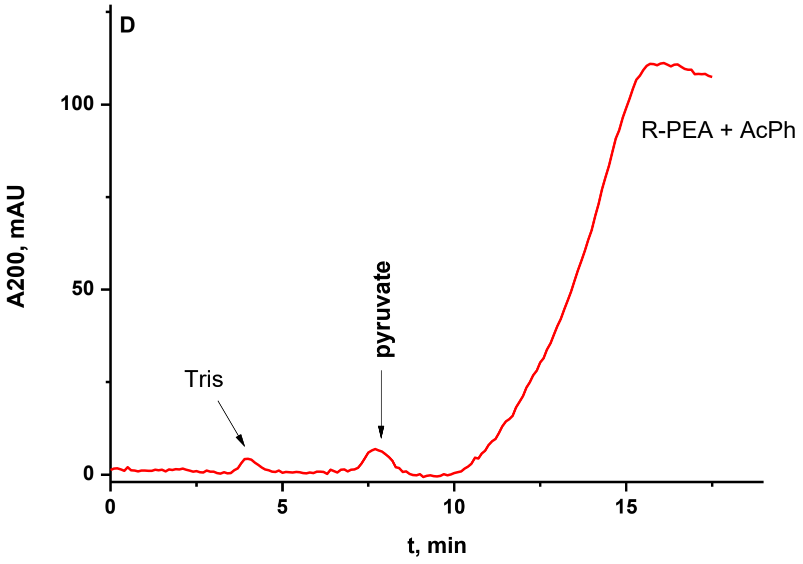
D
A200, mAU
100
50
0
R-PEA + AcPh
pyruvate
Tris
0
5
10
15
t, min
