# Supplementary material for: Probing the role of the residues in the active site of the transaminase from Thermobaculum terrenum
Source: PLoS One. 2021 Jul 29;16(7):e0255098. doi: 10.1371/journal.pone.0255098 (PMC8320979; doi:10.1371/journal.pone.0255098)
Supplement: S1 Table — (PDF) [file pone.0255098.s006.pdf]

Table S1. List of primer sequences for the *TaTT* mutagenesis.

Mutation	Primer	Sequence (from 5’ to 3’ end)
R43S	R43S.R	GTTCCAGTAACC***AGA***GATACCTTCGAAGACCGC
	CheckR43S.F	GCGGTCTTCGAAGGTATC**TC**
Y101F	Y101F.F	ACCGTGGCGATGTG***TTC***ATCATGCCGCTGGC
	CheckY101F.R	*AAG*CCAGCGGCATGAT**GA**
Y166W	Y166W.F	GCATTGGCCAAC***TGG***CGTAATAGCCAGCTG
	CheckY166W.F	AAGGCATTGGCCAAC***TGG***
G41V +R43S	G41V&R43S.F	ACTGCGGTCTTCGAA***GTG***ATC***TCT***GGTTACTGG
	CheckG41V.F	ACTGCGGTCTTCGAA***GTG***
G41V +R43S+F39Y	F39Y&41V&43S.F	ACTGCGGTC***TAC***GAA***GTG***ATC***TCT***GGTTACTGG
	CheckF39Y&G41V.F	ACTGCGGTC**TAC**GAA***GTG***
S115R	S115R.R	TCACCAACAAC***ACG***GAATGCTTTGTTGCCCA
	Check S115R.R	GCGGTCACCAACAAC***ACG***
+A108	108+A.R	GCTTTGTTGCCCAC**GGC***T*GCATAAGCCAGCGGC
	Check108+A.F	GCCGCTGGCTTATGCA**G**
W32H	W32H.F	GACAGACTACTGG***CAT***GCGAGCGTTACTGC
	CheckW32H.F	CTGACAGACTACTGG***CAT***
	universal T7.F	TAATACGACTCACTATAGGG
	universal T7.R	TAGTTATTGCTCAGCGGTGG

Nucleotide substitutions are shown in bold.
