# Supplementary material for: Probing the role of the residues in the active site of the transaminase from Thermobaculum terrenum
Source: PLoS One. 2021 Jul 29;16(7):e0255098. doi: 10.1371/journal.pone.0255098 (PMC8320979; doi:10.1371/journal.pone.0255098)
Supplement: S2 Table — (PDF) [file pone.0255098.s007.pdf]

Table S2. Subfamily-specific position among BCAT and R-TA families of PLP fold type IV.

Subfamily-specific positions are ranked by decreasing statistical significance; higher rank means more significant input of the position on the functional difference between the families. For each position, its "Rank" is indicated (the higher the rank, the more is our interest in the position in terms of its potential influence on function) and specificity estimates (S-, Z-, P-estimates correspond to the initial value of the specificity function, its centered and normalized value and the assessment of statistical reliability, respectively). Positions are numbered according to PDB structure 6GKR. The amino acid content is provided separately for each family in each position.

Rank	S-score	Z-score	P-value	Position in *TaTT* structure (PDB ID 6GKR)	BCATs homologous to TaTT (308 sequences)	R-TAs homologous to TaTT (291 sequences)
1	0.834	6.255	4.94E-08	A/GLY/41	G (99.4%) S (0.3%) - (0.3%)	V (78.7%) T (20.6%) G (0.3%) A (0.3%)
2	0.993	6.075	1.19E-14	A/TYR/166	Y (100.0%)	W (99.3%) R (0.3%) - (0.3%)
3	0.967	5.914	1.18E-20	A/ASN/168	N (96.4%) Q (1.3%) T (1.0%) S (0.6%) L (0.3%) I (0.3%)	D (99.7%) E (0.3%)
4	0.748	5.881	2.68E-27	A/ALA/262	A (65.9%) M (12.7%) V (11.4%) W (3.2%) Y (2.3%) T (1.9%) S (1.0%) G (0.3%) E (0.3%) F (0.3%) C (0.3%) Q (0.3%)	G (99.7%) - (0.3%)
5	0.93	5.861	4.88E-34	A/PHE/39	F (98.7%) Y (1.0%) - (0.3%)	Y (97.6%) Q (2.4%)
6	0.798	5.838	1.06E-40	A/GLY/259	G (100.0%)	T (57.4%) S (41.9%) - (0.3%) A (0.3%)
7	0.783	5.672	9.59E-45	A/GLU/40	E (85.4%) G (11.4%) A (2.6%) - (0.3%) D (0.3%)	D (93.8%) T (5.2%) E (1.0%)
8	0.718	5.604	4.83E-50	A/GLU/263	E (56.5%) Q (33.1%) H (7.5%) G (1.6%) A (0.6%) N (0.6%)	G (99.7%) - (0.3%)
9	0.699	5.57	7.54E-56	A/TRP/148	W (93.5%) F (4.5%) Y (1.3%) I (0.6%)	V (68.7%) T (7.2%) Q (4.5%) P (4.1%) L (3.8%) R (2.4%) I (2.1%) Y (2.1%) E (1.4%) M (1.0%) H (1.0%) S (0.7%) - (0.7%) N (0.3%)
10	0.817	5.519	4.43E-61	A/TYR/45	Y (91.2%) N (8.1%) F (0.4%) - (0.3%)	W (89.7%) R (2.7%) S (2.4%) F (1.7%) V (0.7%) H (0.7%) L (0.7%) K (0.3%) T (0.3%) C (0.3%) Y (0.3%)
11	0.697	5.491	9.07E-67	A/SER/34	Y (86.0%) F (7.1%) W (2.9%) S (2.3%) T (0.6%) R (0.6%) - (0.3%)	H (72.2%) R (26.1%) K (1.4%) W (0.3%)
12	0.636	5.479	8.16E-73	A/SER/169	S (73.4%) G (7.5%) T (7.1%) A (5.8%) N (4.9%) M (0.3%) Y (0.3%) L (0.3%) P (0.3%)	L (76.6%) F (14.1%) M (9.3%)
13	0.598	5.467	7.64E-79	A/GLY/44	A (49.4%) G (36.7%) C (10.4%) S (1.6%) V (1.3%) - (0.3%) T (0.3%)	V (92.8%) I (3.4%) A (2.1%) T (1.0%) F (0.3%) M (0.3%)
14	0.742	5.444	1.85E-84	A/VAL/35	G (94.8%) A (2.3%) V (2.3%) T (0.3%) - (0.3%)	S (92.4%) G (5.2%) A (2.4%)
15	0.578	5.363	6.45E-88	A/SER/199	E (57.1%) A (17.9%) M (13.0%) S (9.4%) C (1.0%) Y (1.0%) G (0.3%) Q (0.3%)	F (86.6%) Y (11.0%) A (1.4%) S (0.7%) C (0.3%)
16	0.71	5.356	7.22E-94	A/THR/36	T (59.4%) L (15.9%) A (7.1%) S (5.8%) N (3.2%) I (1.9%) V (1.6%) F (1.3%) G (1.0%) C (1.0%) W (0.6%) Y (0.3%) - (0.3%) D (0.3%)	D (99.7%) N (0.3%)
17	0.574	5.345	1.20E-99	A/ALA/164	G (64.6%) A (30.5%) S (3.9%) P (1.0%)	H (50.9%) Q (47.8%) M (0.7%) - (0.3%) N (0.3%)
18	0.709	5.295	9.84E-104	A/MET/53	L (62.3%) M (8.8%) I (6.2%) S (5.2%) V (5.2%) A (2.6%) P (2.6%) F (2.3%) T (2.3%) N (1.0%) R (1.0%) H (0.3%) Y (0.3%)	G (98.3%) S (0.7%) N (0.3%) D (0.3%) R (0.3%)

19	0.569	5.258	3.15E-108	A/ARG/43	R (91.6%) P (3.9%) K (3.2%) G (0.3%) A (0.3%) C (0.3%) - (0.3%)	H (36.1%) S (33.3%) A (14.1%) T (6.9%) P (4.8%) G (3.8%) V (0.3%) I (0.3%) N (0.3%)
20	0.605	5.202	1.21E-111	A/ALA/162	A (25.6%) I (24.0%) V (22.1%) C (9.1%) S (5.5%) L (4.5%) P (3.9%) T (2.9%) M (1.6%) N (0.6%)	N (97.3%) H (2.4%) - (0.3%)
21	0.642	5.15	4.66E-115	A/MET/103	R (93.2%) C (2.6%) H (1.6%) V (0.6%) M (0.6%) T (0.3%) Y (0.3%) Q (0.3%) L (0.3%)	E (59.8%) A (16.8%) Q (7.6%) N (4.5%) S (3.1%) - (2.1%) W (2.1%) C (1.0%) M (1.0%) D (0.7%) Y (0.3%) V (0.3%) K (0.3%) L (0.3%)
22	0.833	5.121	1.81E-119	A/PRO/157	P (89.0%) S (5.2%) M (3.6%) V (1.0%) F (0.6%) I (0.3%) A (0.3%)	D (93.1%) N (3.8%) E (1.0%) S (1.0%) - (0.7%) Y (0.3%)
23	0.676	5.07	1.25E-122	A/TYR/107	Y (68.5%) F (28.9%) W (1.0%) C (0.3%) S (0.3%) I (0.3%) L (0.3%) G (0.3%)	T (82.1%) L (6.9%) S (5.8%) A (3.4%) - (1.0%) M (0.3%) V (0.3%)
24	0.673	4.985	9.56E-124	A/TYR/30	H (56.2%) N (11.0%) P (10.1%) K (8.1%) Q (4.5%) A (2.6%) V (2.3%) R (1.3%) T (1.0%) G (0.6%) L (0.6%) I (0.3%) - (0.3%) S (0.3%) Y (0.3%) E (0.3%)	D (97.9%) - (1.0%) E (0.7%) H (0.3%)
25	0.586	4.874	4.25E-123	A/TRP/31	A (61.4%) T (12.7%) G (11.4%) S (9.1%) V (2.9%) F (1.3%) M (0.3%) W (0.3%) C (0.3%) - (0.3%)	F (83.5%) L (8.2%) Y (6.9%) V (1.0%) - (0.3%)
26	0.485	4.753	1.33E-121	A/PRO/104	P (83.1%) Q (6.5%) V (2.9%) L (1.9%) I (1.6%) M (1.6%) A (1.3%) T (1.0%)	M (45.0%) L (33.0%) I (13.4%) V (3.4%) F (2.4%) W (2.1%) - (0.7%)
27	0.395	4.717	1.30E-124	A/ILE/42	I (76.6%) L (10.7%) V (5.8%) M (2.9%) T (2.3%) F (1.0%) - (0.3%) E (0.3%)	V (45.4%) P (36.8%) A (10.7%) I (3.8%) T (1.7%) - (1.4%) F (0.3%)
28	0.653	4.663	1.96E-126	A/GLY/297	G (89.0%) N (3.6%) A (3.2%) S (1.9%) C (1.6%) D (0.3%) R (0.3%)	H (71.5%) R (9.3%) E (6.2%) D (2.7%) L (2.1%) A (2.1%) Y (1.7%) M (1.4%) S (0.7%) N (0.7%) Q (0.7%) I (0.7%) K (0.3%)
29	0.43	4.662	2.98E-131	A/LEU/171	L (76.9%) F (10.1%) M (6.5%) V (2.6%) I (2.6%) Y (1.0%) A (0.3%)	R (53.3%) K (10.3%) A (10.0%) M (8.6%) Q (4.5%) S (3.4%) L (3.4%) T (2.7%) D (0.7%) I (0.7%) C (0.7%) N (0.7%) G (0.7%) E (0.3%)
30	0.445	4.594	5.76E-132	A/ILE/55	I (31.8%) V (30.8%) L (15.3%) A (14.6%) G (4.2%) M (1.0%) T (0.6%) C (0.6%) F (0.3%) S (0.3%) Y (0.3%)	F (88.0%) I (5.8%) L (3.1%) Y (1.7%) V (0.7%) T (0.3%) A (0.3%)
31	0.453	4.505	4.93E-131	A/ILE/102	I (64.6%) V (16.9%) L (12.3%) M (4.5%) F (1.0%) Y (0.3%) A (0.3%)	V (95.9%) C (1.7%) I (1.0%) - (0.7%) L (0.3%) A (0.3%)
32	0.549	4.407	2.26E-129	A/ALA/108	K (44.5%) V (14.6%) F (8.4%) L (6.5%) R (5.2%) I (4.9%) Y (3.9%) T (3.6%) N (2.9%) A (2.6%) P (1.9%) D (0.3%) S (0.3%) G (0.3%)	R (99.0%) - (1.0%)
33	0.477	4.399	2.93E-133	A/VAL/38	V (62.0%) C (21.4%) I (9.1%) A (3.9%) L (1.9%) F (0.6%) T (0.3%) - (0.3%) Y (0.3%)	T (86.6%) V (5.5%) A (4.1%) C (3.4%) F (0.3%)
34	0.433	4.285	4.67E-130	A/VAL/100	V (39.6%) T (16.6%) C (13.3%) I (13.0%) L (7.1%) A (5.5%) F (1.3%) M (1.0%) S (1.0%) G (1.0%) D (0.6%)	A (86.6%) S (10.3%) V (1.7%) G (1.0%) - (0.3%)
35	0.574	4.228	1.96E-130	A/ARG/292	F (52.9%) A (7.1%) L (6.8%) D (6.5%) S (4.9%) E (4.2%) R (3.6%) H (2.9%) M (2.9%) Q (2.6%) Y (2.3%) I (1.0%) N (1.0%) T (0.6%) K (0.3%) V (0.3%)	W (95.5%) F (3.8%) Y (0.7%)

36	0.355	4.162	4.26E-130	A/LEU/163	T (36.0%) G (18.5%) S (13.3%) A (12.7%) C (7.5%) N (4.9%) V (2.6%) F (1.9%) I (1.3%) L (0.6%) Q (0.6%)	Y (44.7%) L (39.9%) F (14.8%) - (0.3%) C (0.3%)
37	0.402	4.139	1.69E-132	A/SER/147	S (88.0%) T (6.5%) N (2.6%) A (1.6%) R (0.6%) K (0.6%)	R (46.7%) D (19.6%) S (10.0%) E (8.2%) K (5.8%) Q (2.7%) N (2.4%) G (1.7%) A (1.4%) P (1.0%) T (0.3%)
38	0.338	4.128	1.04E-135	A/GLN/170	A (50.6%) R (19.2%) I (7.5%) S (6.2%) V (5.8%) Q (4.2%) C (2.3%) G (2.3%) H (0.6%) L (0.6%) T (0.3%) M (0.3%)	T (49.1%) V (37.8%) I (6.9%) E (2.4%) K (2.1%) N (1.7%)
