# Supplementary material for: Probing the role of the residues in the active site of the transaminase from Thermobaculum terrenum
Source: PLoS One. 2021 Jul 29;16(7):e0255098. doi: 10.1371/journal.pone.0255098 (PMC8320979; doi:10.1371/journal.pone.0255098)
Supplement: S3 Table — (PDF) [file pone.0255098.s008.pdf]

Table S3. Superposition of the *TaTT* subunit with the closest homologs.

	**Protein (PDB code)**	Aligned residues, %	**RMSD, Å**	Sequence identity, %
1	Amino acid aminotransferase from *Burkholderia pseudomallei* (4WHX)	96	1.46	37
2	Branched-chain amino acid aminotransferase from *Thermus thermophiles* (2EJ3)	96	1.39	40
3	Branched-chain amino acid aminotransferase from *Thermoproteus uzoniensis* (5CE8)	95	1.33	43
4	Branched-chain amino acid aminotransferase from *E. coli* (1I1K)	94	1.33	37
5	Branched-chain amino acid aminotransferase from *Geoglobus acetivorans* (5E25)	98	1.43	31
6	(*R*)-selective amine transferase from *Aspergillus fumigatus* (4CHI)	98	1.64	24
7	D-amino acid aminotransferase from *Bacillus* sp.YM-1 (1DAA)	93	1.69	30
8	(*R*)-amine transaminase from *Arthrobacter* SP. KNK168 (3WWH)	82	1.65	26
