# Supplementary material for: Probing the role of the residues in the active site of the transaminase from Thermobaculum terrenum
Source: PLoS One. 2021 Jul 29;16(7):e0255098. doi: 10.1371/journal.pone.0255098 (PMC8320979; doi:10.1371/journal.pone.0255098)
Supplement: S4 Table — (PDF) [file pone.0255098.s009.pdf]

Table S4. RMSD (Å) between Cα atoms of subunits of WT *TaTT* and its variants.

TaTT	WT	mP3	mP3O3
WT (PDB ID 6GKR)	-	0.267	0.286
mP3 (PDB ID 7NEA)	0.267	-	0.190
mP3O3 (PDB ID 7NEB)	0.286	0.190	-
