# Supplementary material for: Probing the role of the residues in the active site of the transaminase from Thermobaculum terrenum
Source: PLoS One. 2021 Jul 29;16(7):e0255098. doi: 10.1371/journal.pone.0255098 (PMC8320979; doi:10.1371/journal.pone.0255098)
Supplement: S5 Table — (PDF) [file pone.0255098.s010.pdf]

Table S5. Specificity determining sequence motifs of *TaTT* and TAs from the families of PLP fold type IV.

	**Sequence motif 1**	**Sequence motif 2**	**numbering corresponds to:**
BCAT	^{31}YxxxxF[ED]Gx[KR]^{40}	^{95}YxR^{97}… ^{107}[LMVI]G[VL]^{109}	BCAT from *Escherichia coli*
DAAT	^{26}FxxxxYxV[IVA][KR]^{35}	^{86}HxY^{88}…^{98}[RK]xH^{100}	DAAT from *Bacillus sp*. YM-1
R-TA	^{53}HxxxxYD[VT]x[STAHP]^{62}	^{113}[FY]V[EQAWNS]^{115} … ^{126}[RKFGP]x[STANER]^{128}	R-TA from *Nectria haematococca*
TaTT	^{34}SxxxxFEGIR^{43}	^{101}YIM^{103}………^{114}FSV^{116}	BCAT from *Thermobaculum terrenum*
