# Supplementary material for: Probing the role of the residues in the active site of the transaminase from Thermobaculum terrenum
Source: PLoS One. 2021 Jul 29;16(7):e0255098. doi: 10.1371/journal.pone.0255098 (PMC8320979; doi:10.1371/journal.pone.0255098)
Supplement: S1 File — (PDF) [file pone.0255098.s011.pdf]

The determination of the initial activity of WT *TaTT*, variants mP1 and mO1 in the overall transamination reaction with L-leucine and α-ketoglutarate

Glutamate dehydrogenase (GDH) coupled assay

GDH calibration curve.

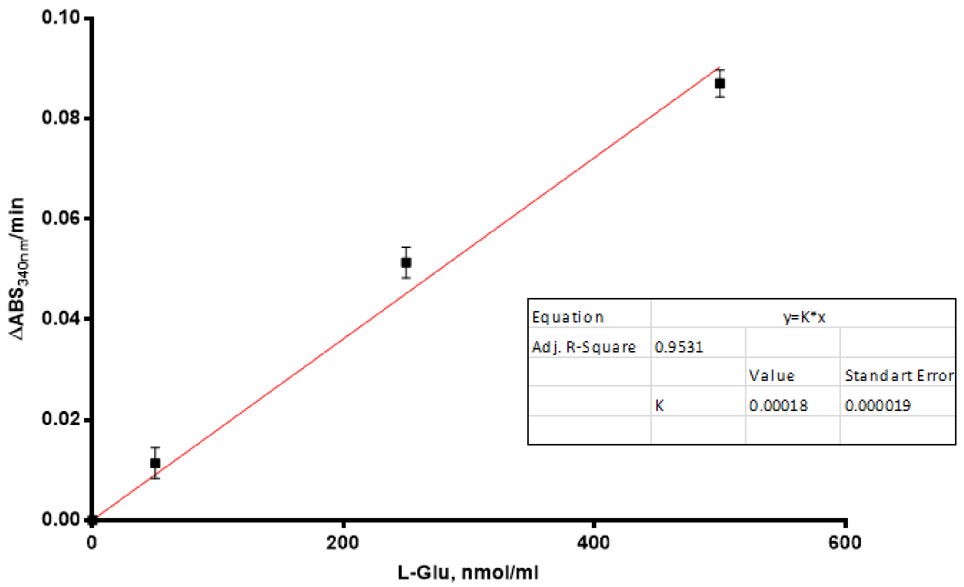

GDH calibration curve represents the change of absorbance at 340 nm per minute corresponding NADH formation associated with L-glutamate deamination by glutamate dehydrogenase (GDH). Reaction conditions: 50 mM Tris-HCl pH 9.0, 1 mM NAD^+, (10 μl of tenfold diluted GDH (Sigma-Aldrich, USA, № G2626)) in a final volume 1 mL at 25 °C. Reaction was initiated by the addition of L-glutamate of known concentration. In GDH coupled assay the reaction was initiated by adding 100 μL aliquot of a reaction mixture.

The initial activity of WT *TaTT* and its variants in the overall reaction was measured using GDH-coupled assay to assess L-glutamate production. Appropriate amounts of enzyme [6.5 μg of WT *TaTT* (pH 8.0), 12 μg of WT *TaTT* (pH 7.0) ,400 μg of variant mP1 and 160 μg of variant mO1] was added into 1 mL reaction mixture, containing 50 mM Tris, pH 8.0 (or pH 7.0), 60 μM PLP,100 mM NaCl, 1 mM α-ketoglutarate, 5 mM L-Leu at 50°C. 250 μL aliquots were taken at time intervals of 1,2,3,4,5 minutes and flash-freezed in a liquid nitrogen to stop the reaction. Accumulation of L-glutamate was measured by the protocol above. Raw data indicating the change of absorbance at 340 nm per minute vs time of the transamination reaction and the amount of the produced L-glutamate per mg of the enzyme *vs*. time of the reaction are presented.

For **WT *TaTT (at pH 8.0).***

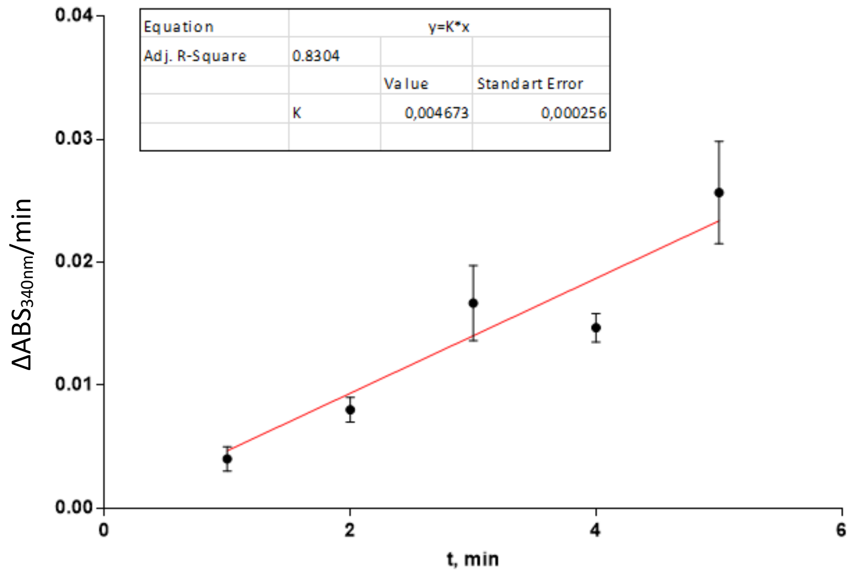

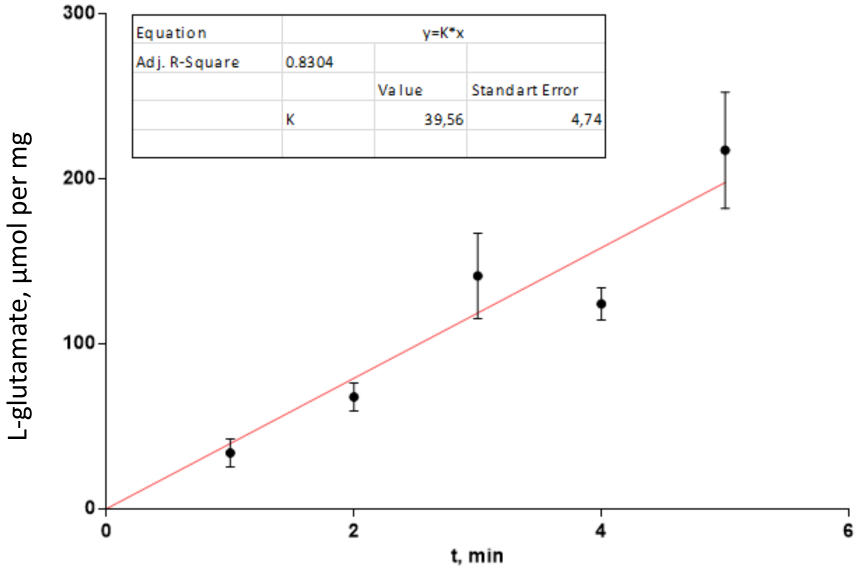

For **WT *TaTT (at pH 7.0).***

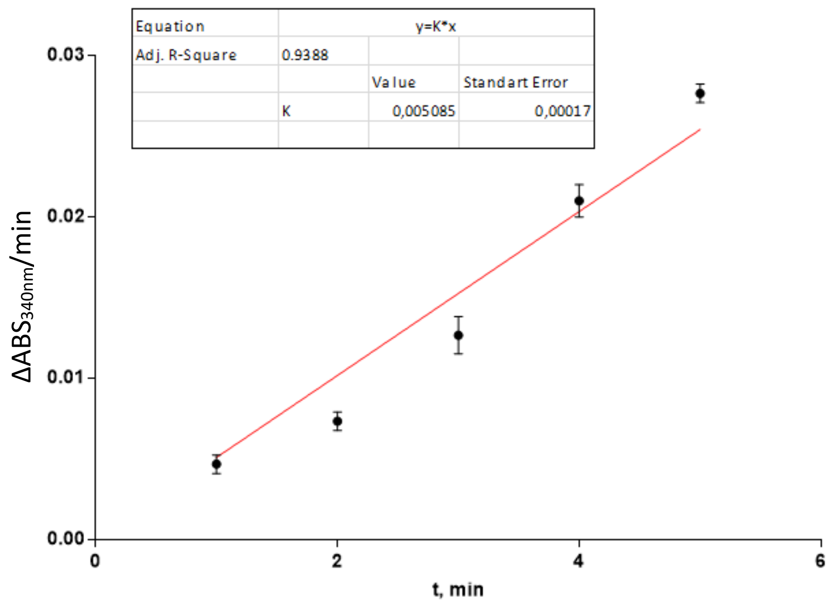

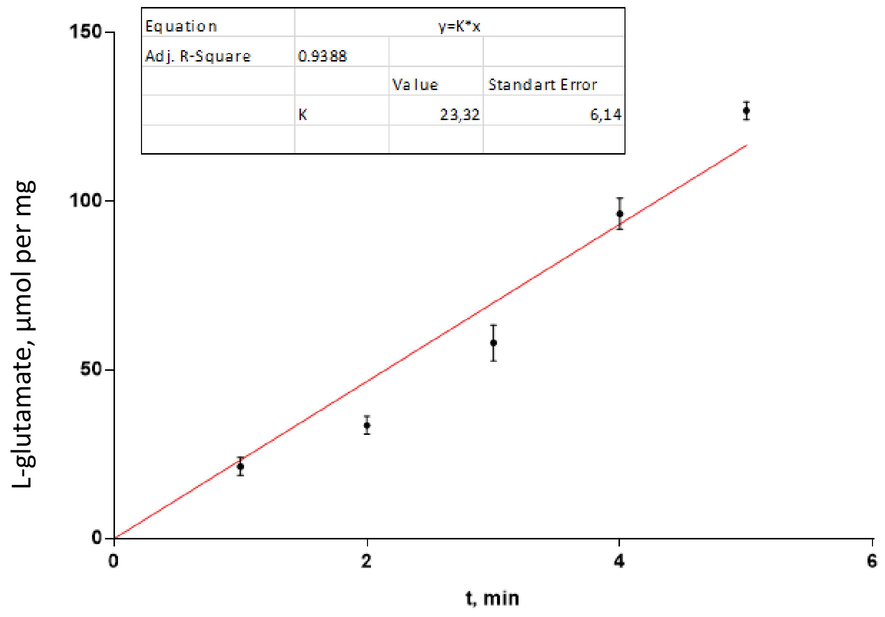

For **mP1**

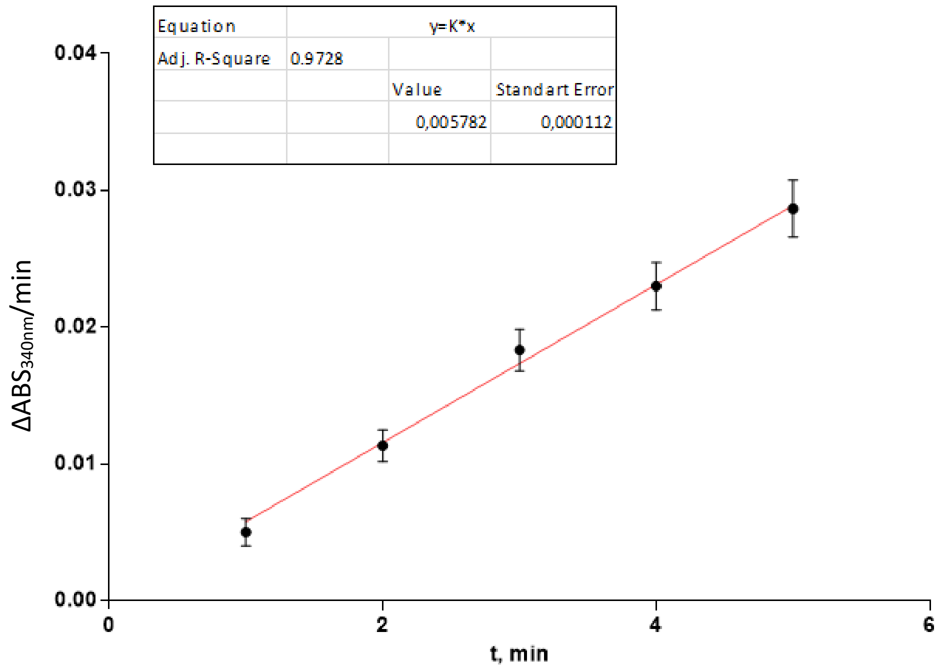
Equation
y=K*x
Adj. R-Square
0.9728
Value
Standart Error
0,005782
0,000112
ΔABS340nm/min
t, min
0.04
0.03
0.02
0.01
0.00
0
2
4
6

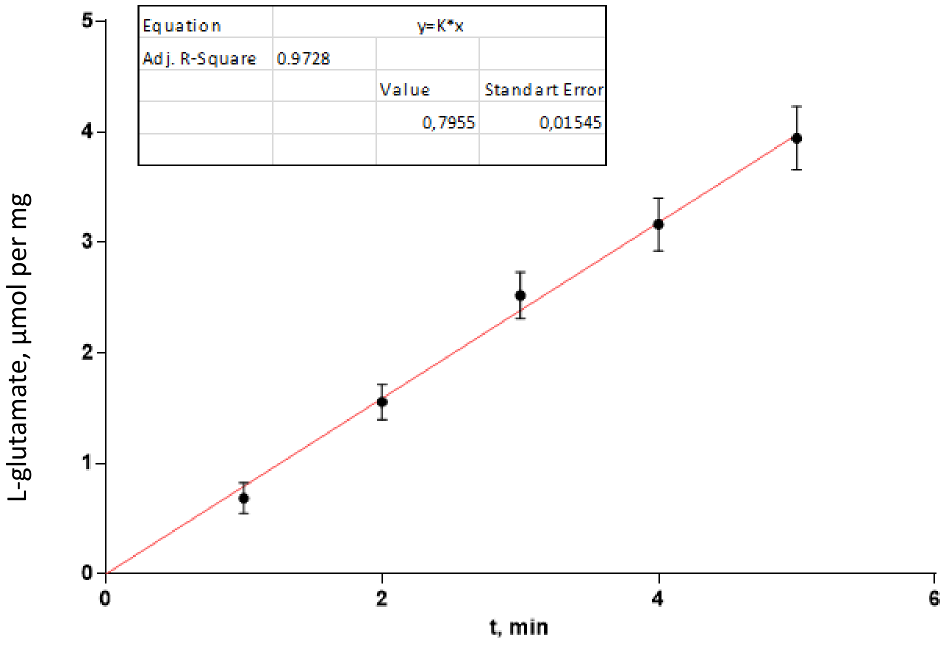
Equation
y=K*x
Adj. R-Square
0.9728
Value
Standart Error
0,7955
0,01545
L-glutamate, µmol per mg
t, min
5
4
3
2
1
0
0
2
4
6

For **mO1**

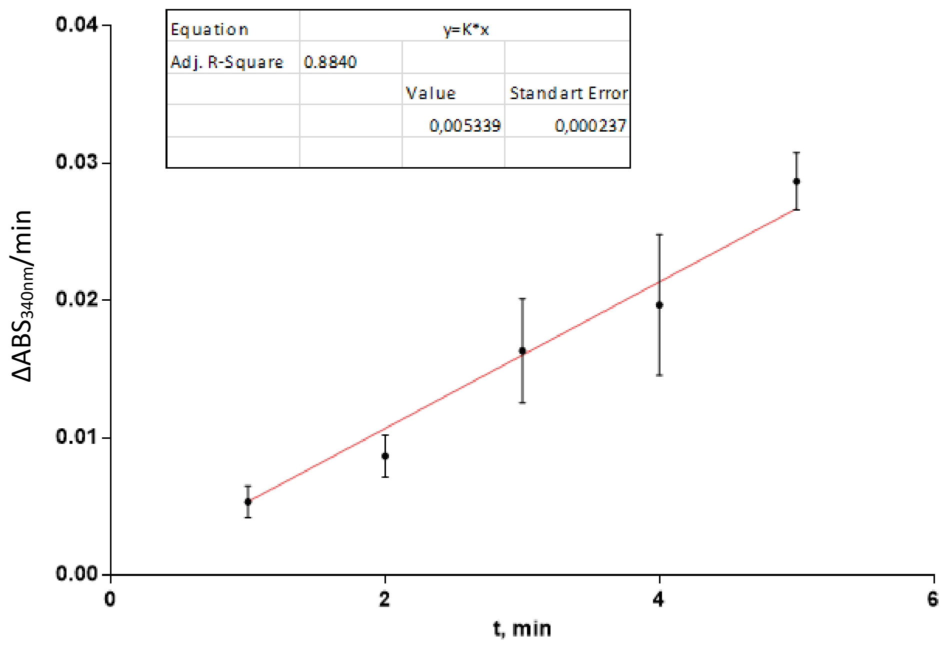
Equation
y=K*x
Adj. R-Square
0.8840
Value
Standart Error
0,005339
0,000237
0.04
0.03
0.02
0.01
0.00
ΔABS340nm/min
0
2
4
6
t, min

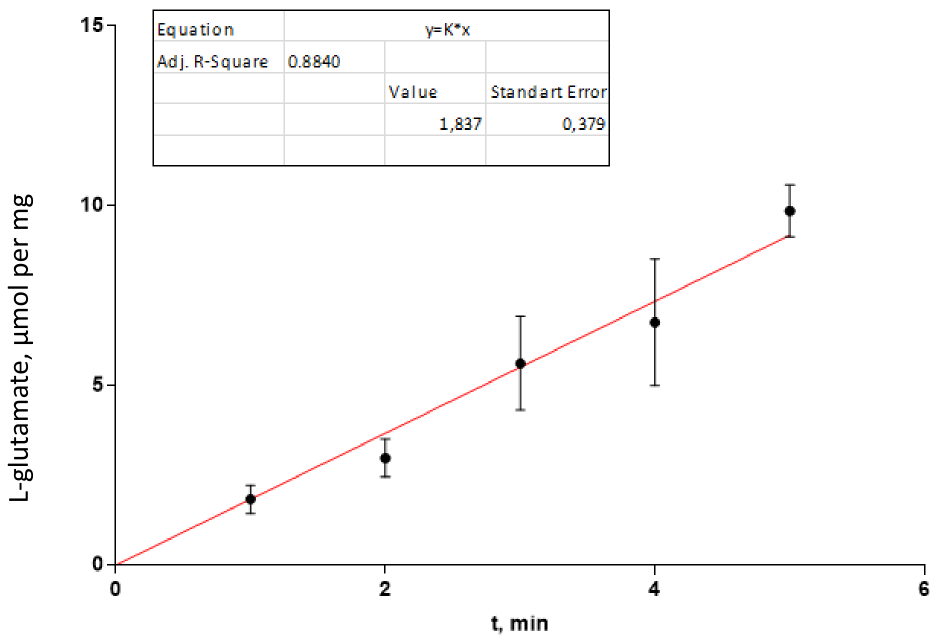
Equation
y=K*x
Adj. R-Square
0.8840
Value
Standart Error
1,837
0,379
15
10
5
0
L-glutamate, µmol per mg
0
2
4
6
t, min
