# Supplementary material for: Probing the role of the residues in the active site of the transaminase from Thermobaculum terrenum
Source: PLoS One. 2021 Jul 29;16(7):e0255098. doi: 10.1371/journal.pone.0255098 (PMC8320979; doi:10.1371/journal.pone.0255098)

The melting temperature measurements

The unfolding curves were obtained by subtracting the average fluorescence intensity values of buffer from the average fluorescence values of samples, containing the protein. The curves were fitted with Boltzmann model. The melting temperature (Tm) was identified as the midpoint of the protein unfolding transition.

Experimental conditions: The samples diluted to 0.5 mg/ml in 50 mM sodium phosphate buffer, pH 8.0, containing 200 mM NaCl, 20 µM PLP and 1000x dilution of Sypro Orange dye (Sigma-Aldrich), were transferred to a 96-well PCR plates (Thermo Fischer Scientific) in the final volume 50 µl per well. Buffer mixed with Sypro dye was used as a negative control. Plates were placed in a real-time cycler (Bio-Rad C1000, with the CFX96 Real-Time accessory, USA), and the temperature was ramped up from 20 to 95 °C in 1.0 °C min-1. Relative fluorescence was measured using the FRET channel, and GraphPad Prism v8.0 (GraphPad Software Inc., USA) was used to fit the collected data a sigmoidal curve and calculate Tm using Boltzmann model. Assays were run in triplicate, and error bars correspond to the standard deviation of the mean (some are too small to be seen).

For WT *TaTT*

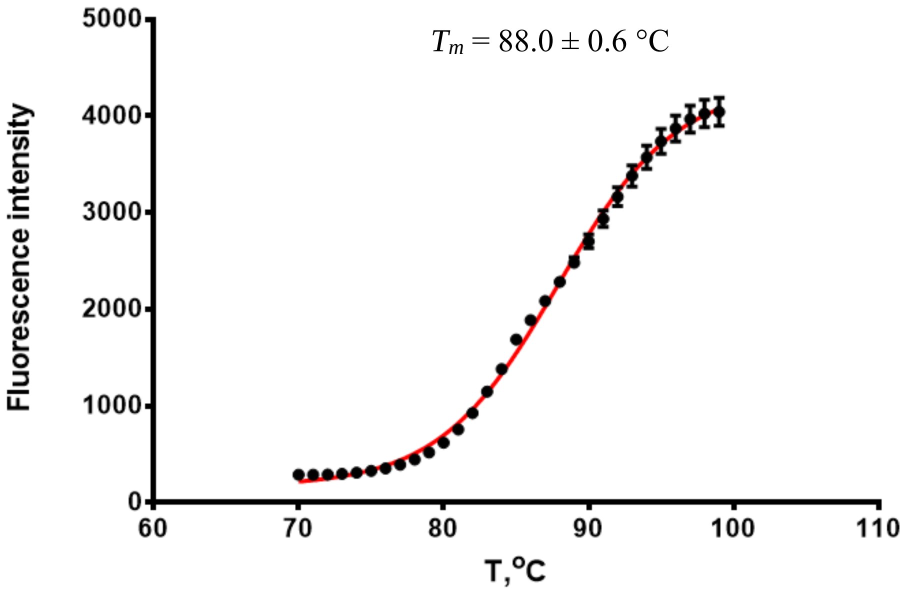

For mP1 variant

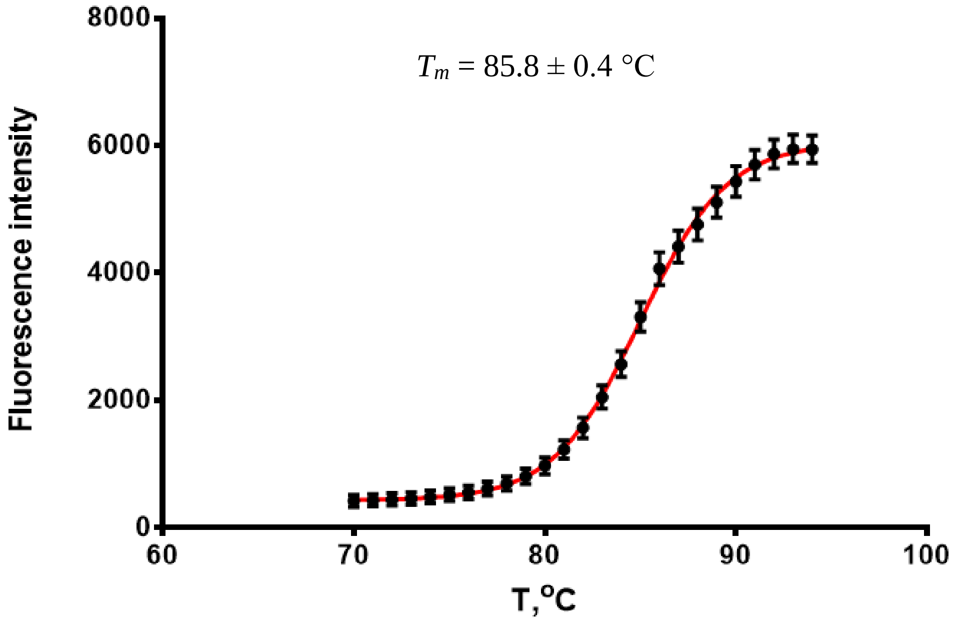

For mP2 variant

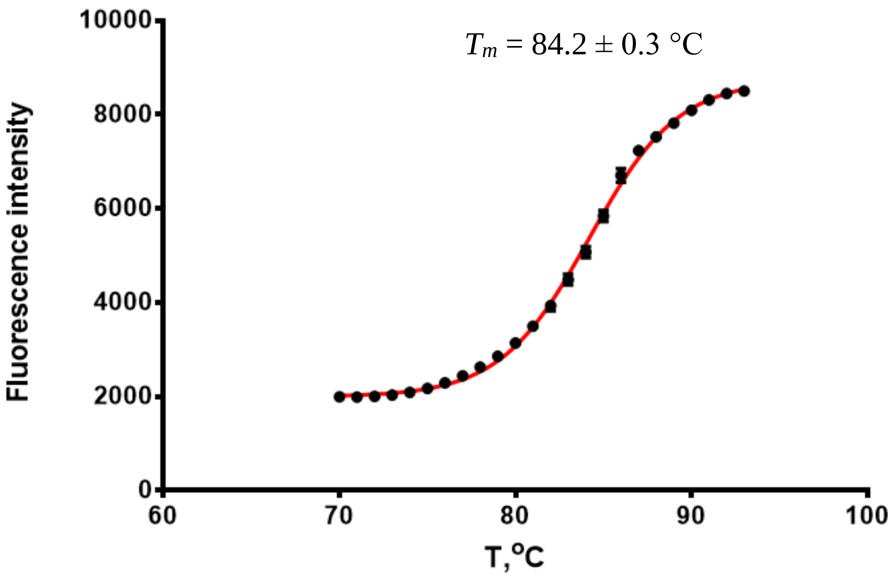

For mP3 variant

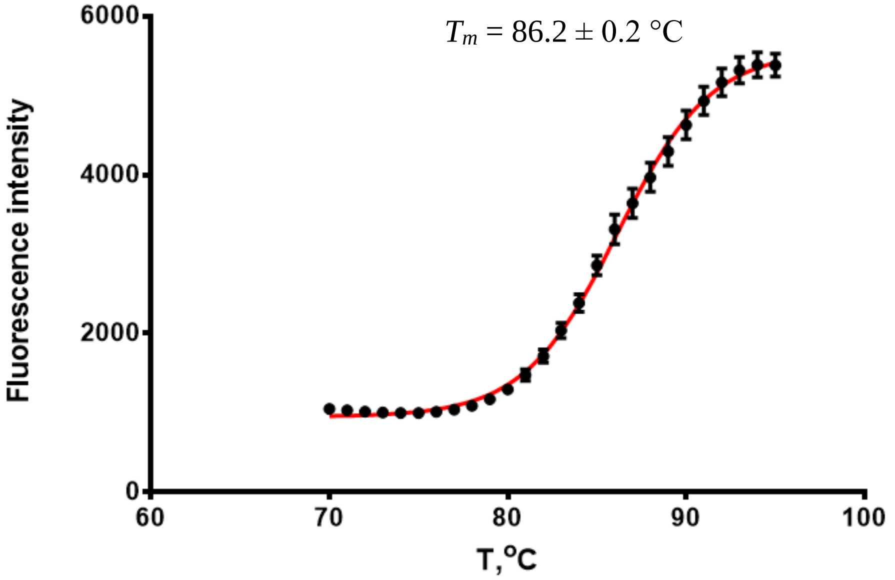

For mO2 variant

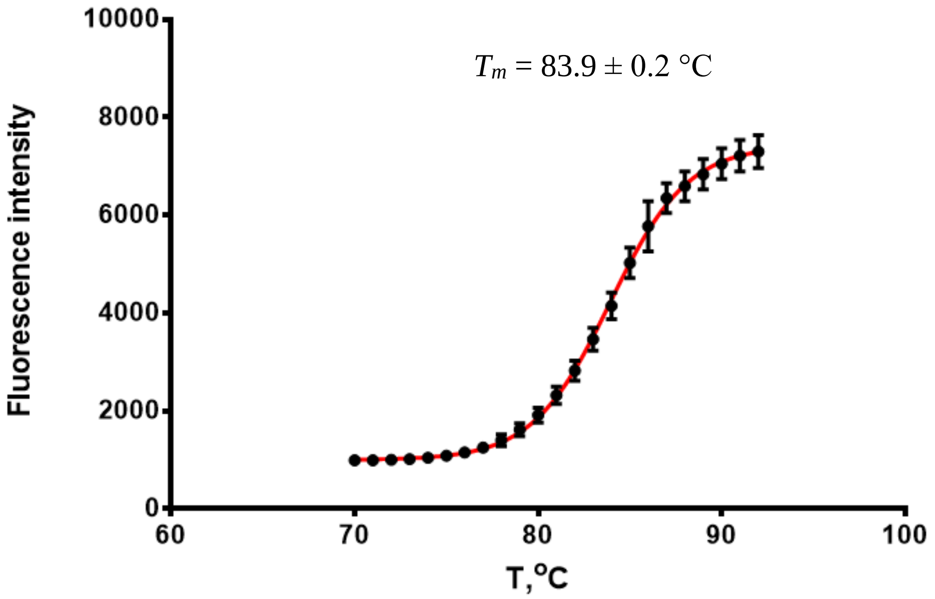

For mP3O1 variant

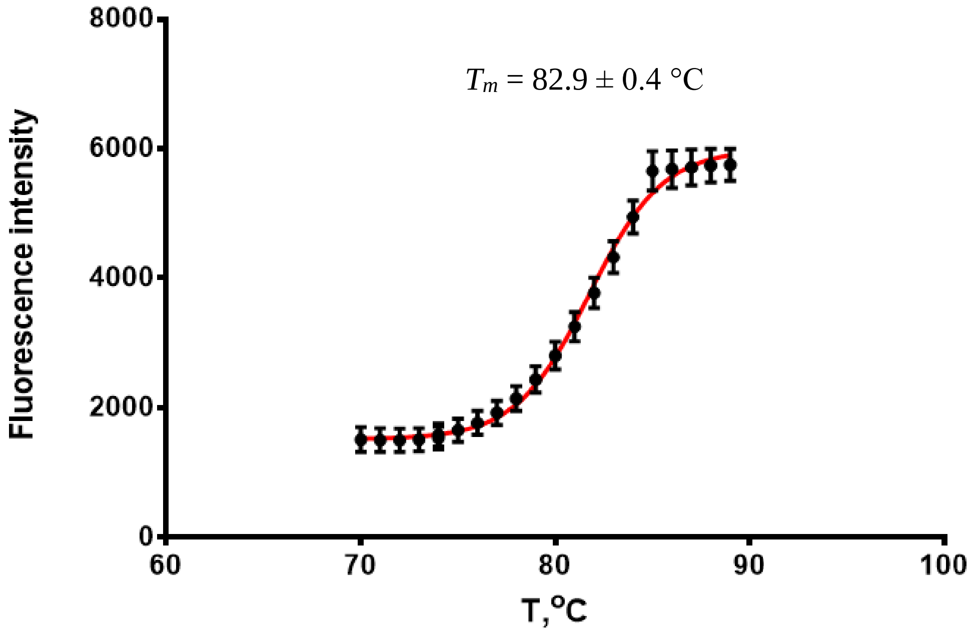

Supplement: S2 File — (PDF) [file pone.0255098.s012.pdf]
