# Supplementary material for: Probing the role of the residues in the active site of the transaminase from Thermobaculum terrenum
Source: PLoS One. 2021 Jul 29;16(7):e0255098. doi: 10.1371/journal.pone.0255098 (PMC8320979; doi:10.1371/journal.pone.0255098)

The dependence of the observed rate constant of half-reaction (k_{obs}) on concentration of ((*R*)-(+)-1-phenylethylamine (R-PEA) in 50 mM CHES buffer, pH 9.0, at 40 °C. All data are collected in triplicate, and error bars correspond to the standard deviation of the mean (some are too small to be seen).

For **WT *TaTT***

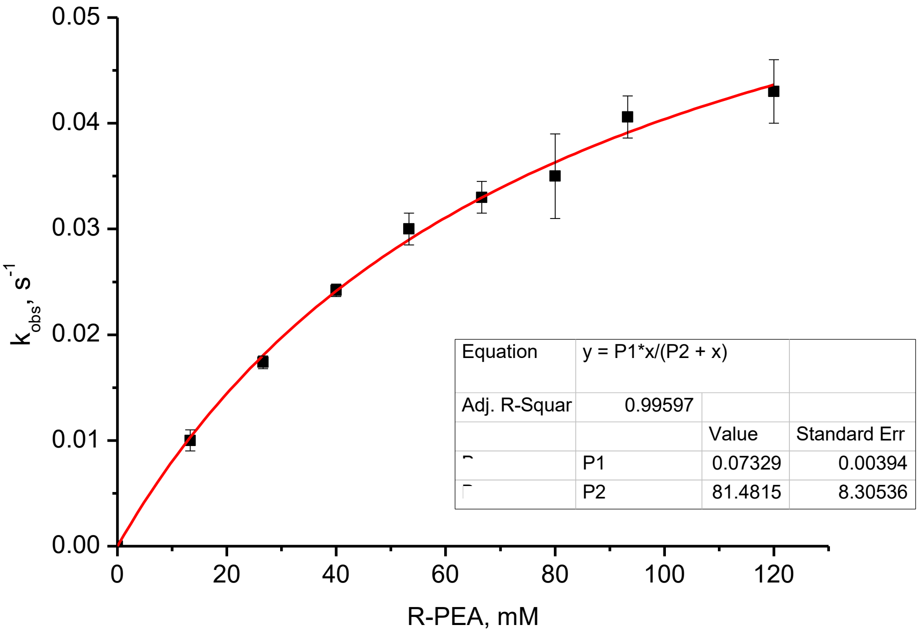

For **mP2** variant

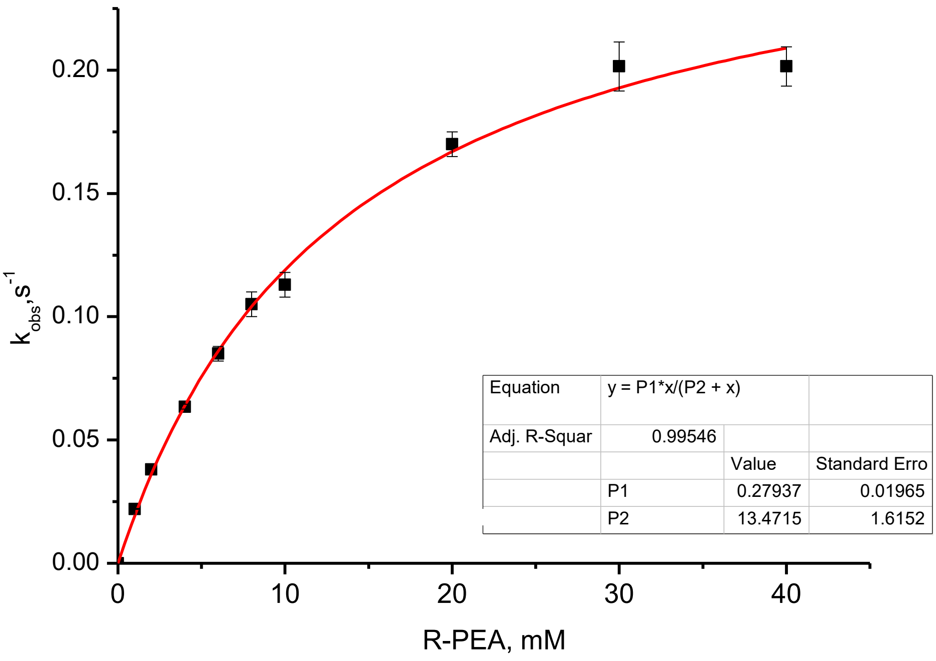

For **mP3** variant

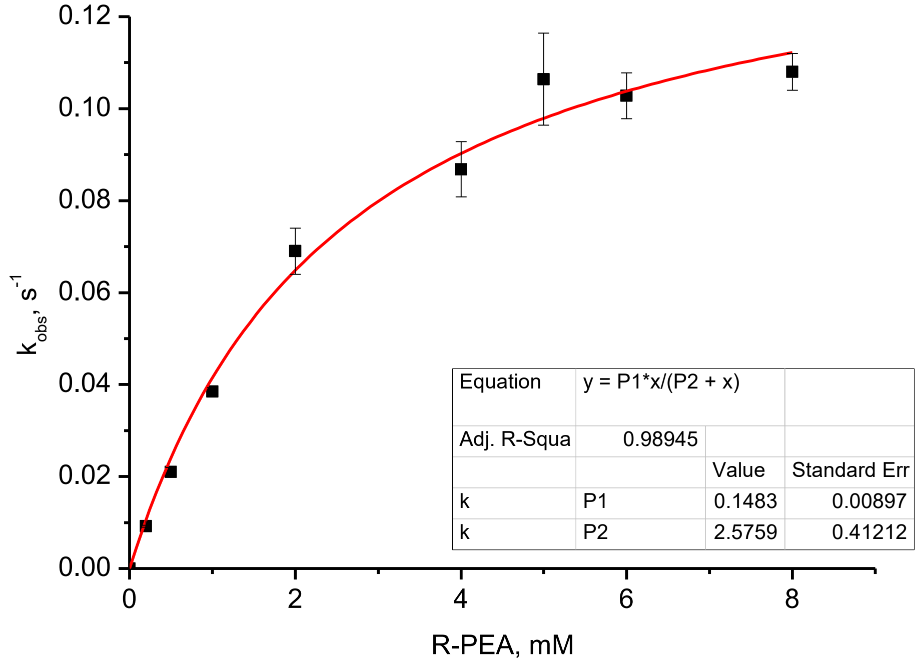

For **mP3O1** variant

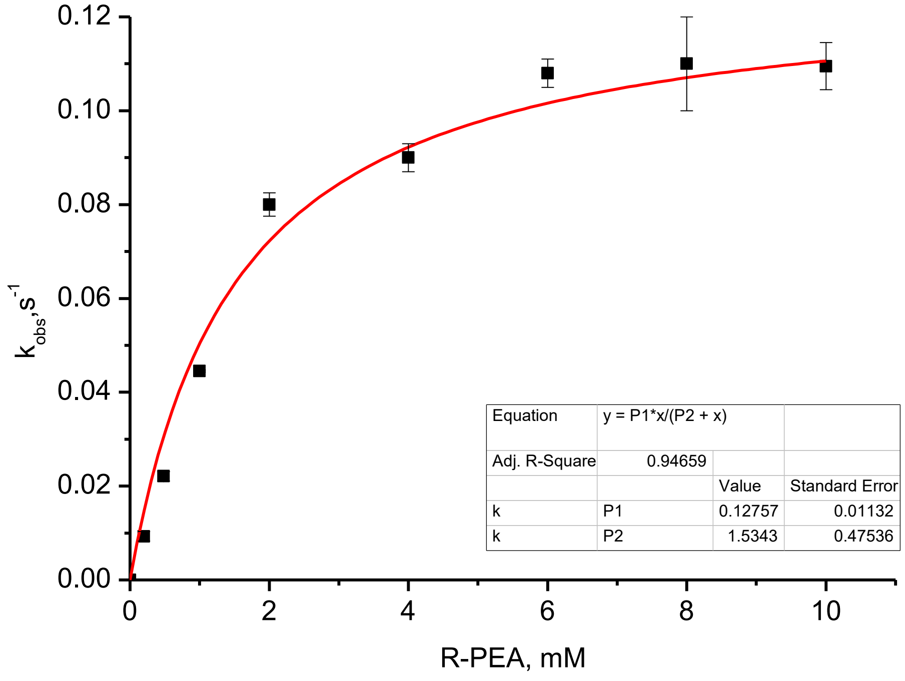

For **mO1**

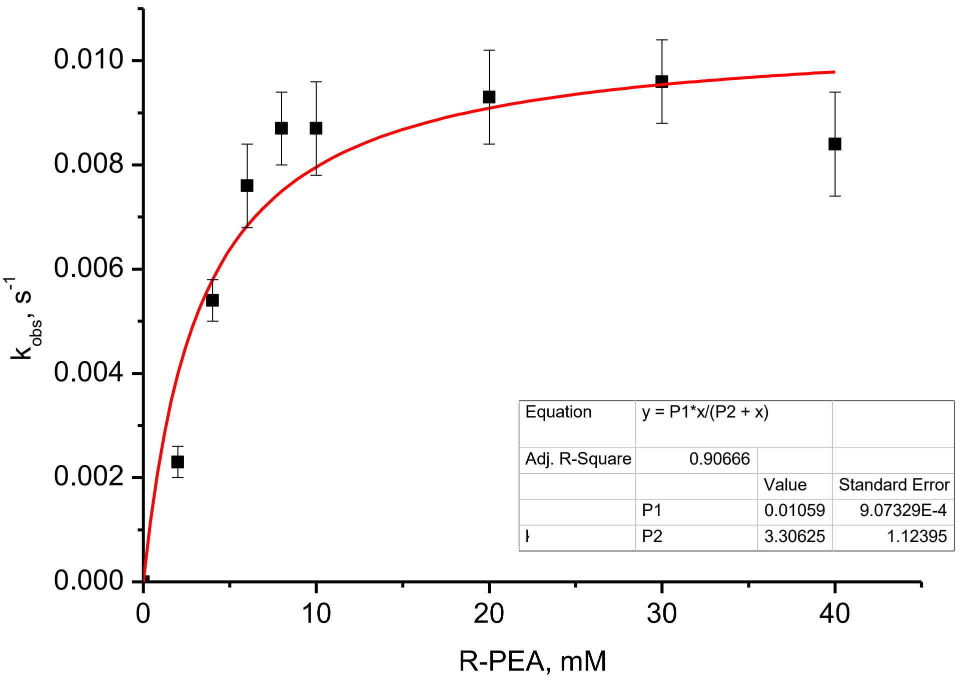
0.010
0.008
0.006
0.004
0.002
0.000
k_obs, s^-1
0
10
20
30
40
R-PEA, mM
Equation
y = P1*x/(P2 + x)
Adj. R-Square
0.90666
Value
Standard Error
P1
0.01059
9.07329E-4
P2
3.30625
1.12395

The dependence of the observed rate constant of half-reaction (k_{obs}) on concentration of ((*R*)-(+)-1-phenylethylamine (R-PEA) in 50 Tris-HCl buffer, pH 8.0, at 30 °C

For **WT *TaTT***

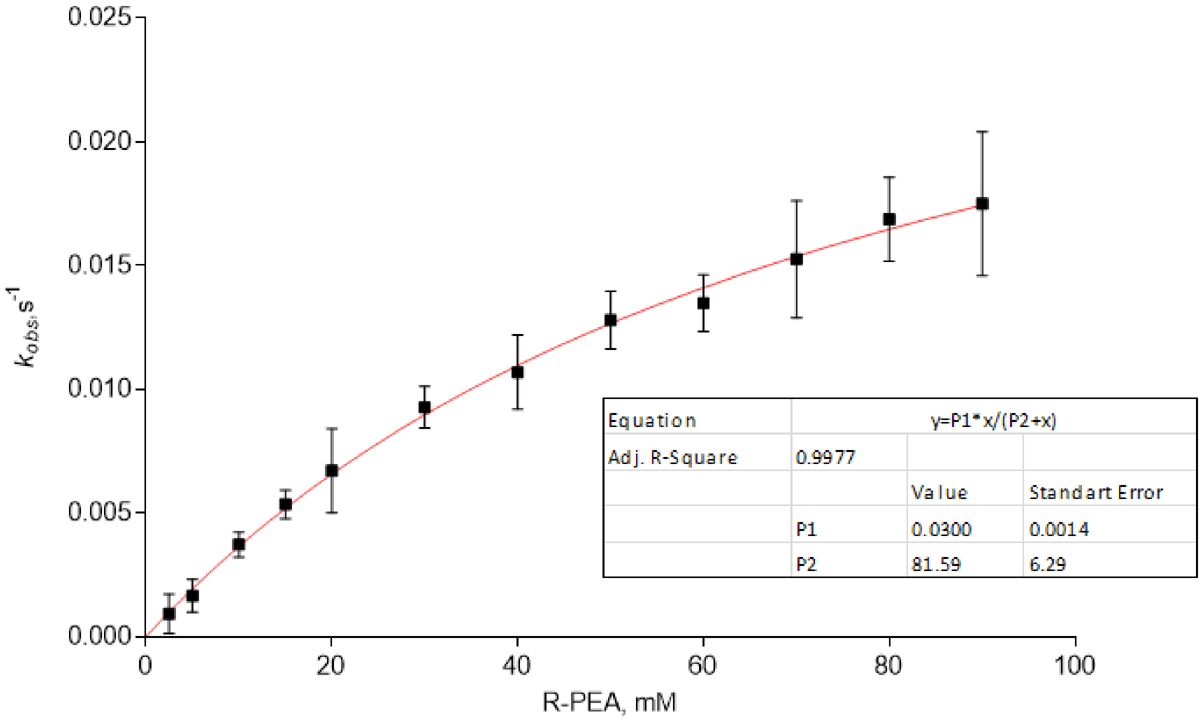

For **mP1**

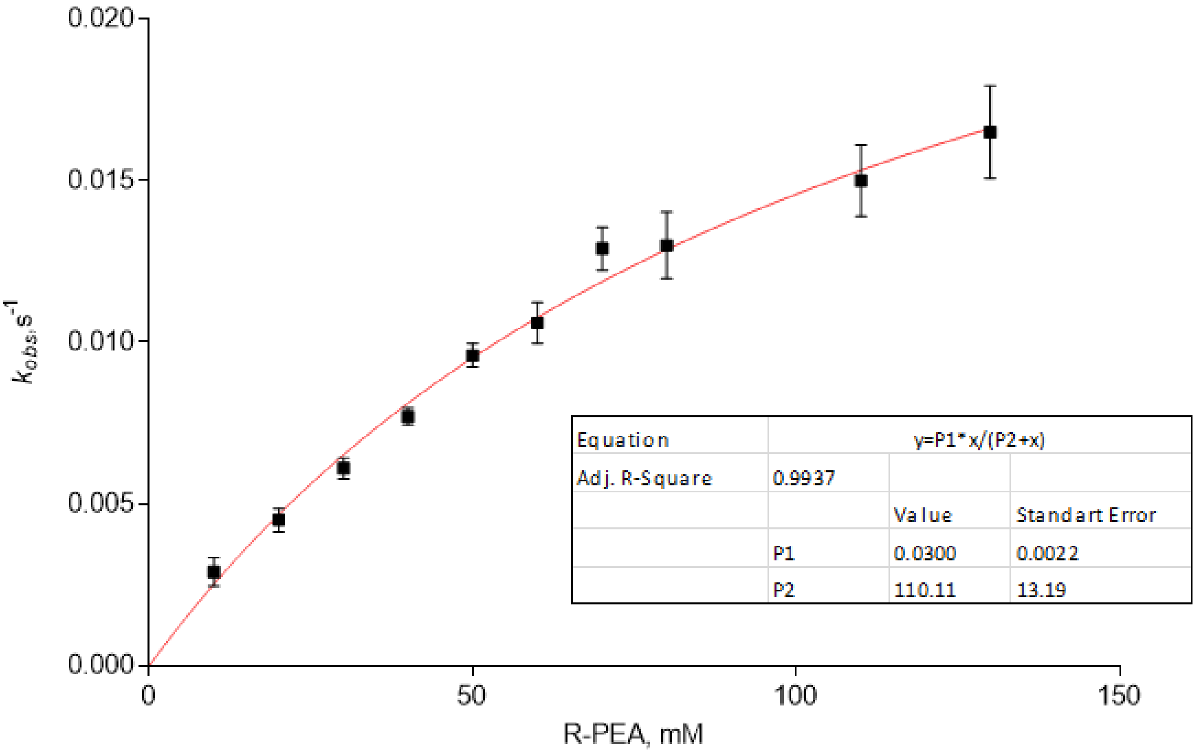

For **mP2**

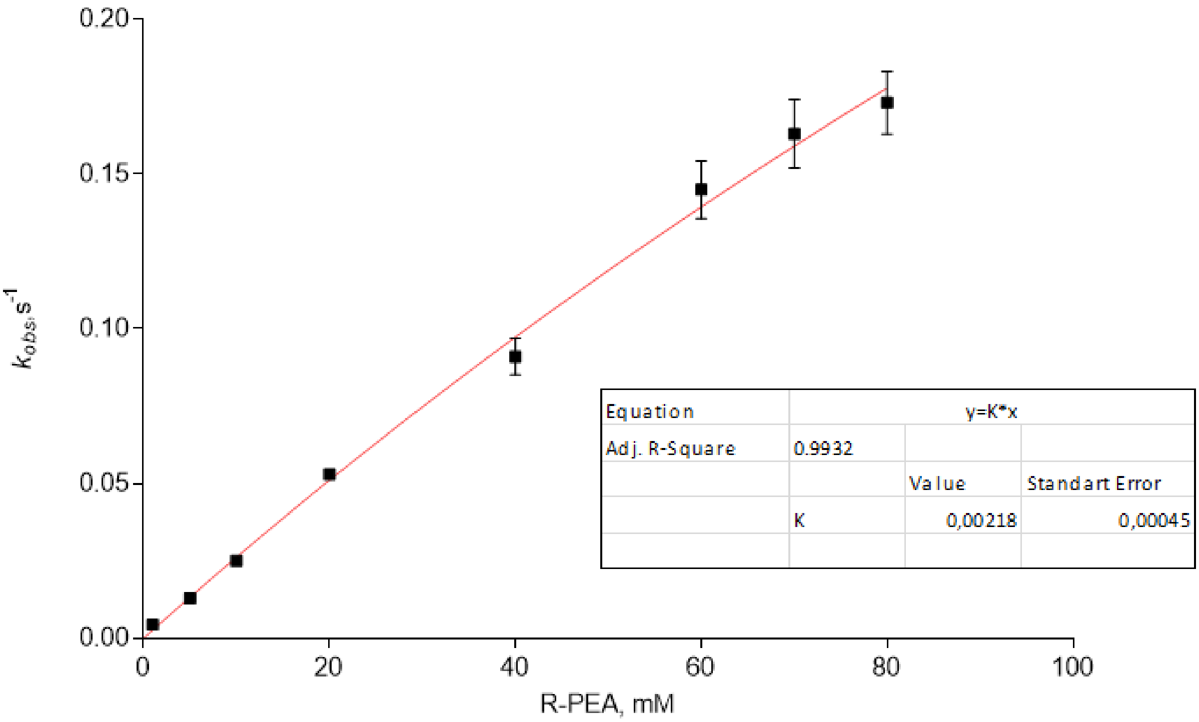

For **mP3**

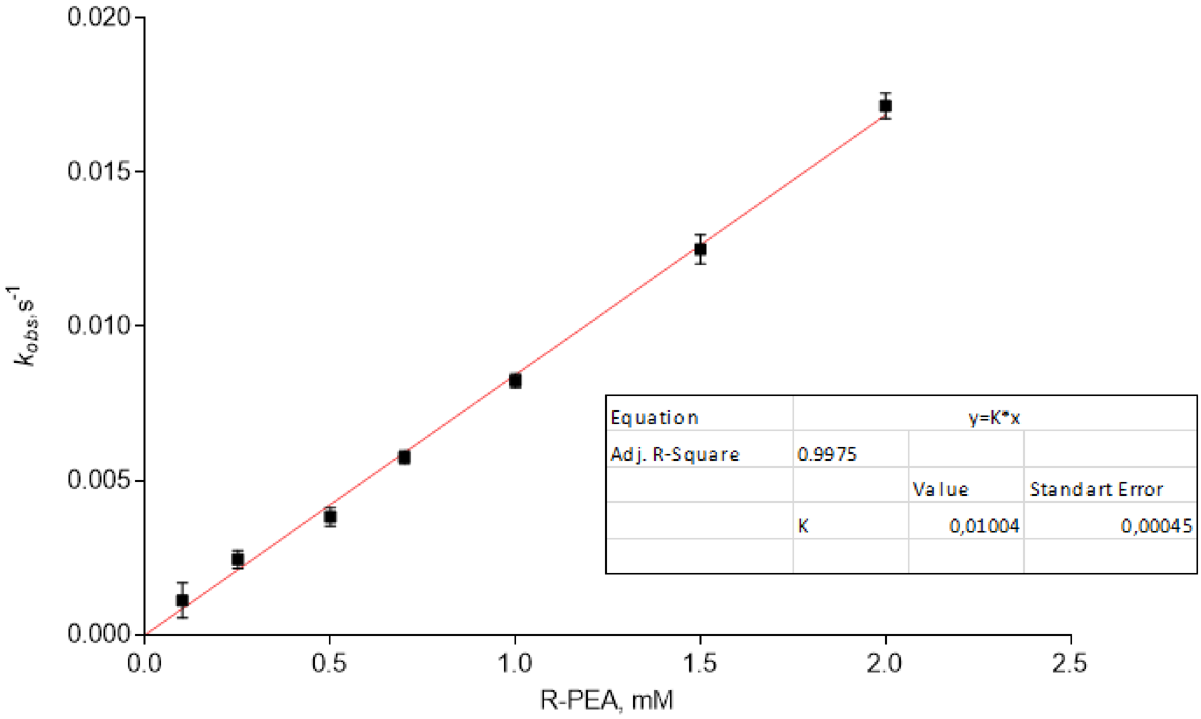

For **mO1**

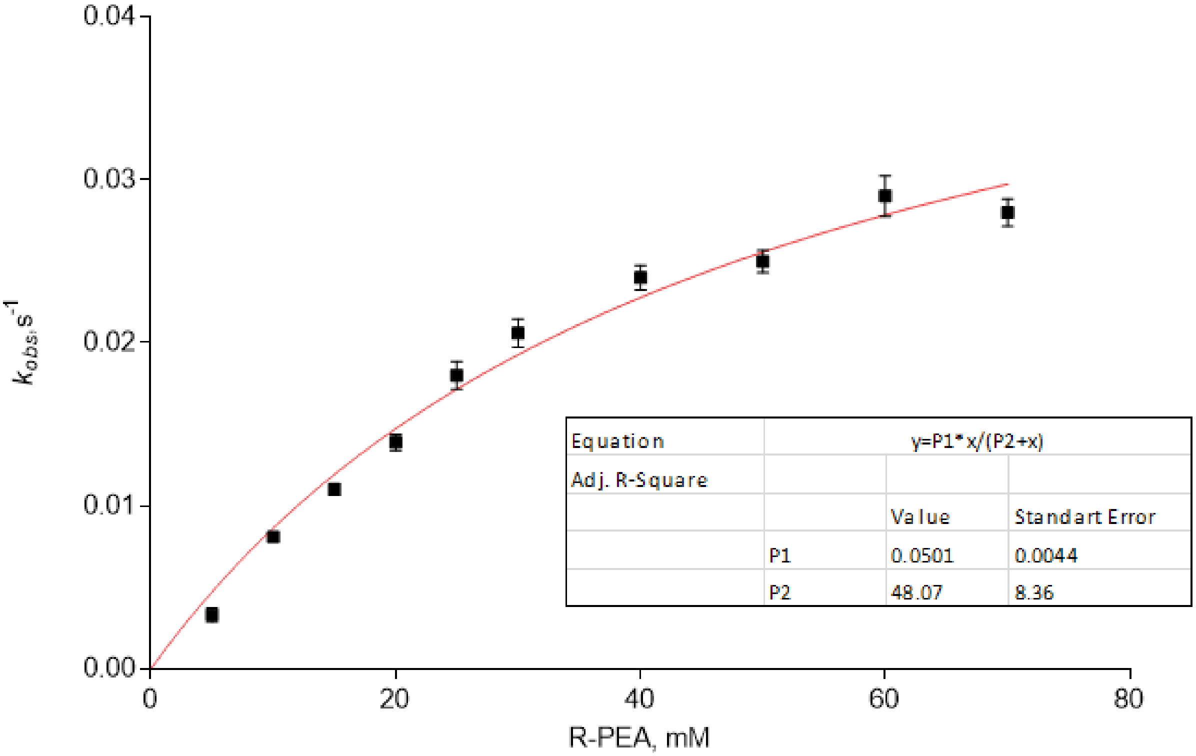

For **mP2O1**

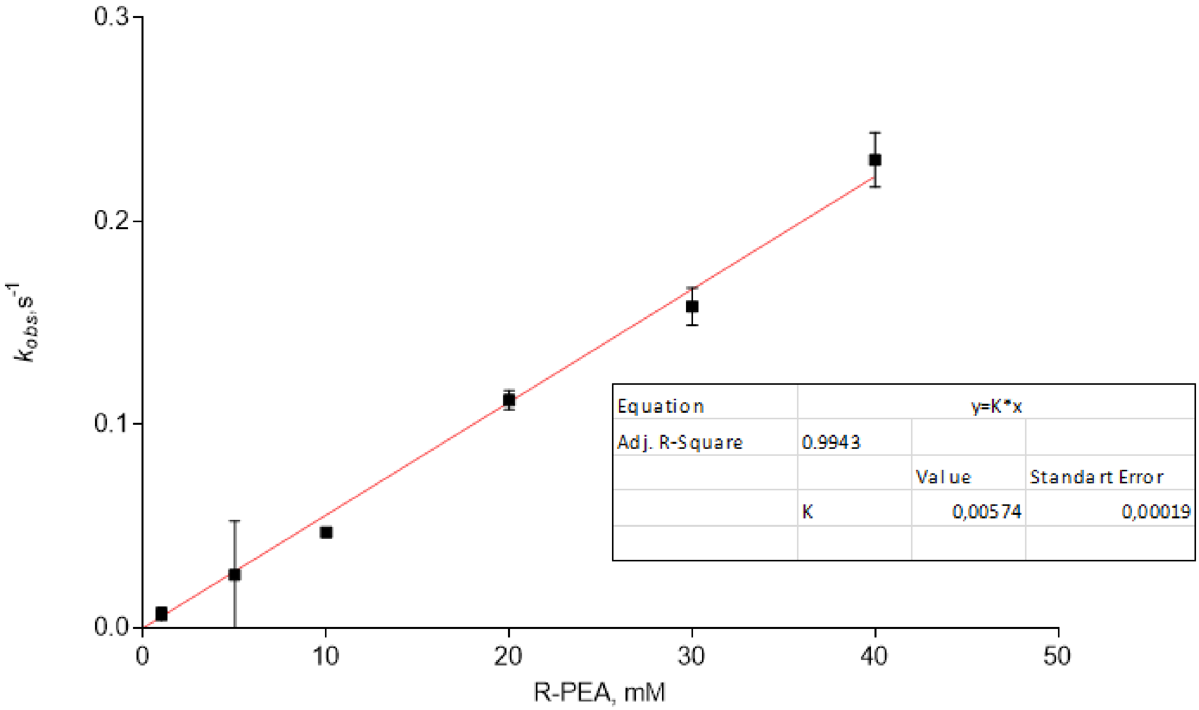

For **mP3O1**

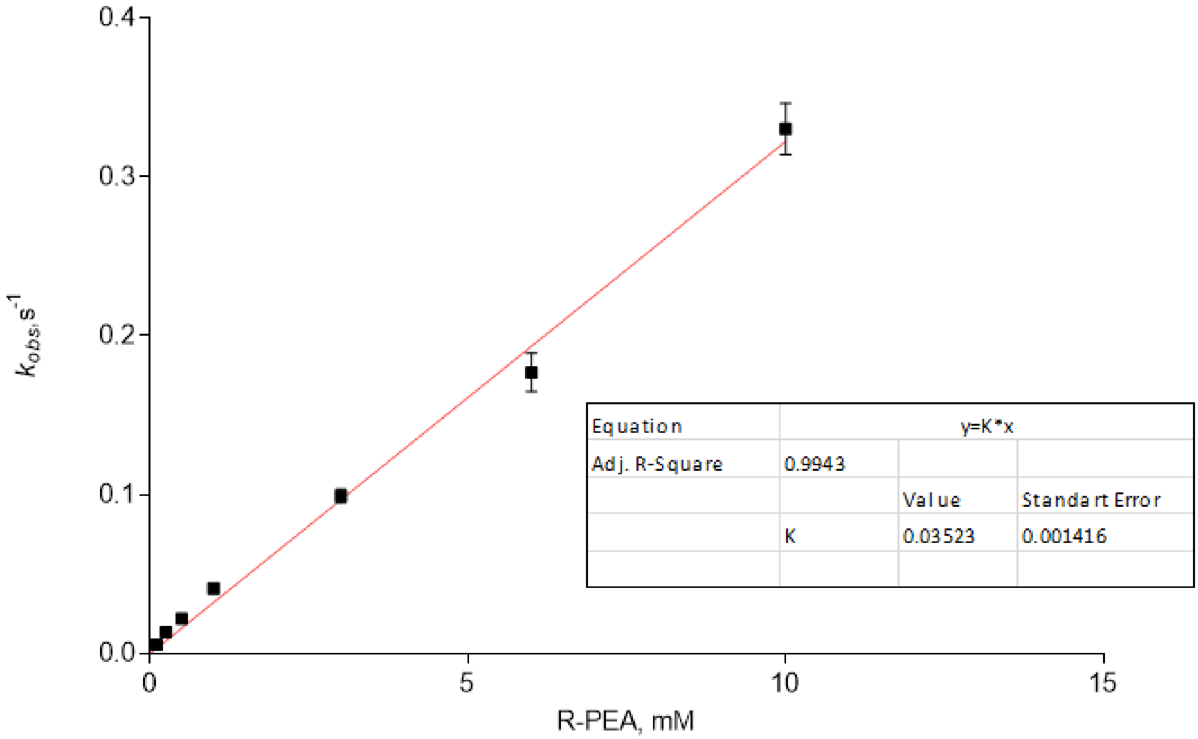

For **mP3O3**

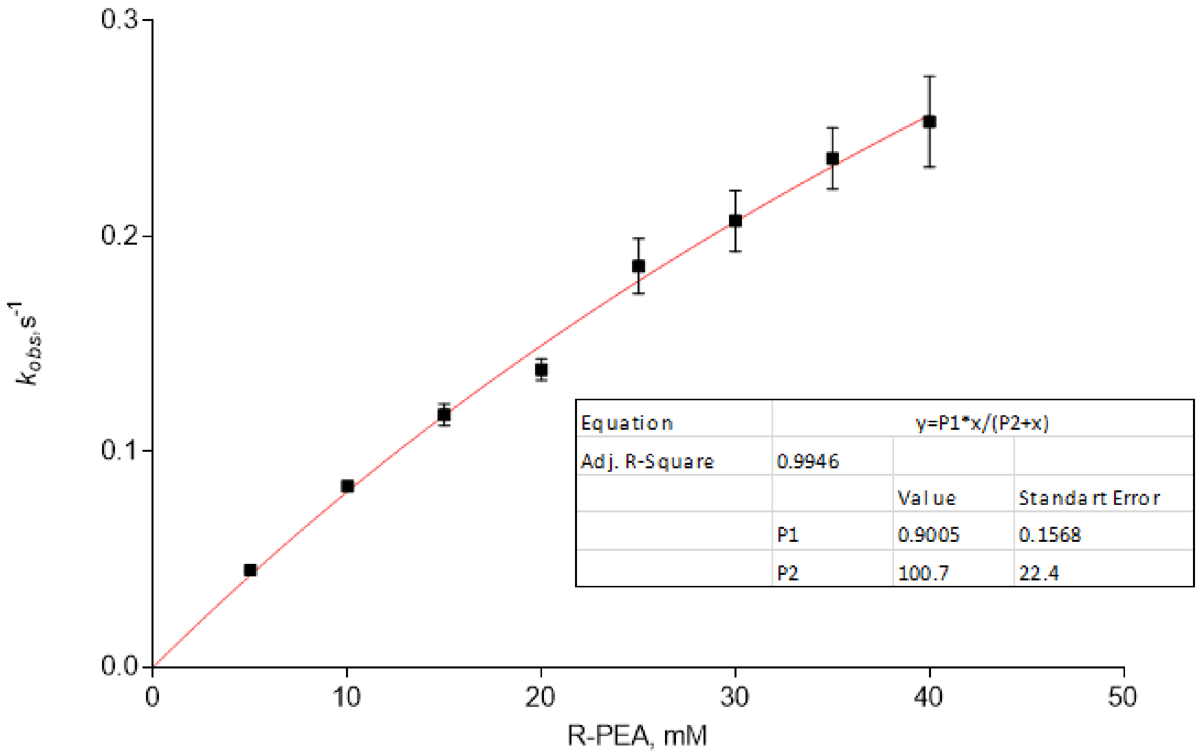

For **mP3O5**

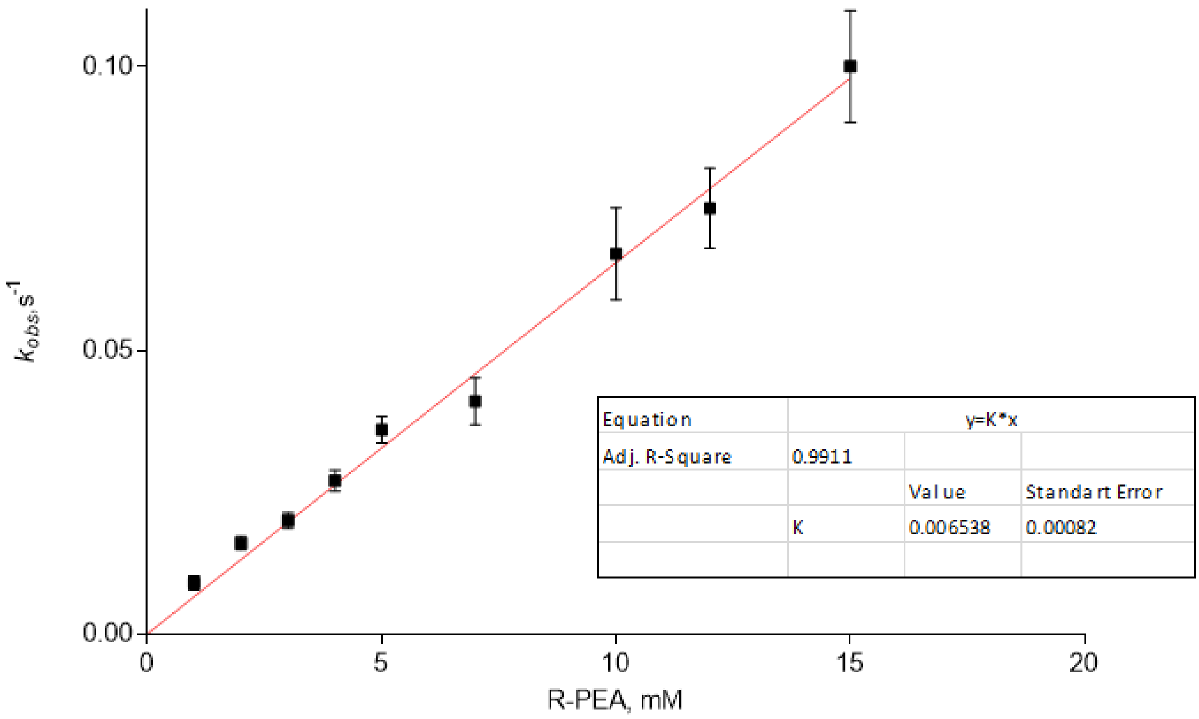

For **mP3O7**

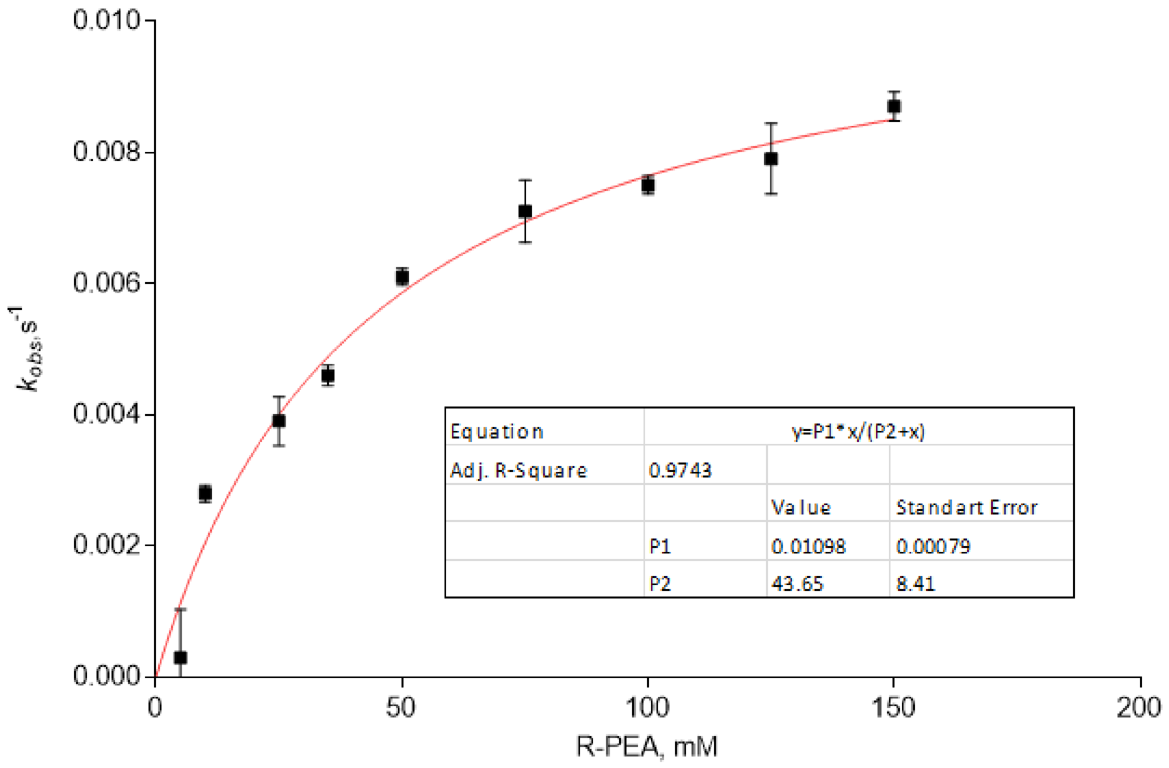
0.010
0.008
0.006
0.004
0.002
0.000
k_{obs}, s^{-1}
0
50
100
150
200
R-PEA, mM
Equation
y=P1*x/(P2+x)
Adj. R-Square
0.9743
Value
Standart Error
P1
0.01098
0.00079
P2
43.65
8.41

The dependence of the observed rate constant of half-reaction (k_{obs}) on concentration of ((*R*)-(+)-1-phenylethylamine (R-PEA) in 50 mM Tris-HCl buffer, pH 7.0, at 40 °C. All data are collected in triplicate, and error bars correspond to the standard deviation of the mean (some are too small to be seen).

For **WT *TaTT***

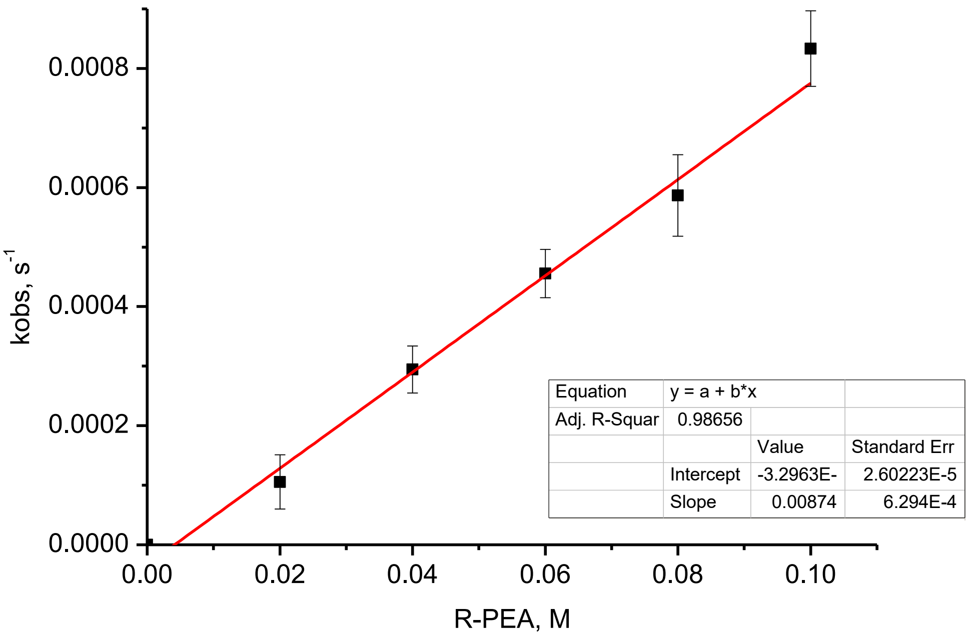

For **mP3O1** variant

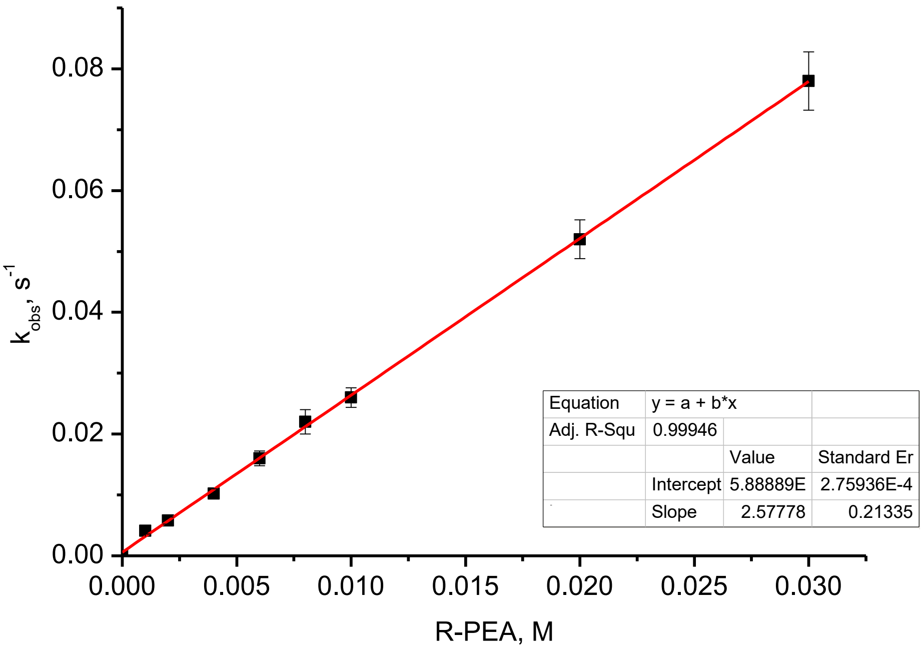

Supplement: S3 File — (PDF) [file pone.0255098.s013.pdf]
